# New Chalcone–Triazole Hybrids with Promising Antimicrobial Activity in Multidrug Resistance Strains

**DOI:** 10.3390/ijms232214291

**Published:** 2022-11-18

**Authors:** Daniela Pereira, Fernando Durães, Nikoletta Szemerédi, Joana Freitas-da-Silva, Eugénia Pinto, Paulo Martins-da-Costa, Madalena Pinto, Marta Correia-da-Silva, Gabriella Spengler, Emília Sousa, Honorina Cidade

**Affiliations:** 1 Laboratory of Organic and Pharmaceutical Chemistry, Department of Chemical Sciences, FFUP—Faculty of Pharmacy, University of Porto, 4050-313 Porto, Portugal; 2 CIIMAR—Interdisciplinary Centre of Marine and Environmental Research, University of Porto, 4450-208 Matosinhos, Portugal; 3 Department of Medical Microbiology, Albert Szent-Györgyi Health Center and Albert Szent-Györgyi Medical School, University of Szeged, 6725 Szeged, Hungary; 4 ICBAS—Institute of Biomedical Sciences Abel Salazar, University of Porto, 4050-313 Porto, Portugal; 5 Laboratory of Microbiology, Department of Biological Sciences, Faculty of Pharmacy, University of Porto, 4050-313 Porto, Portugal

**Keywords:** chalcones, 1,2,3-triazole, antibacterial, efflux pump inhibition

## Abstract

Resistance to antibiotics is an emerging problem worldwide, which leads to an increase in morbidity and mortality rates. Several mechanisms are attributed to bacterial resistance, overexpression of efflux pumps being one of the most prominent. As an attempt to develop new effective antimicrobial drugs, which could be able to act against resistant bacterial strains and considering the antimicrobial potential of flavonoids and triazolyl flavonoid derivatives, in particular chalcones, a small library of chalcone derivatives was synthesized and evaluated for its potential to act as antimicrobials and/or adjuvants in combination with antibiotics towards resistant bacteria. Although only compound **7** was able to act as antibacterial, compounds **1**, **2**, **4**, **5**, **7,** and **9** revealed to be able to potentiate the activity of antibiotics in resistant bacteria. Moreover, five compounds (**3**, **5**–**8**) demonstrated to be effective inhibitors of efflux pumps in *Salmonella enterica* serovar Typhimurium SL1344, and four compounds (**1**, **3**, **7,** and **10**) showed higher ability than reserpine to inhibit biofilm formation of resistant *Staphylococcus aureus* 272123. Together, our results showed the potential of these compounds regarding reversion of bacterial resistance.

## 1. Introduction

Multidrug resistance (MDR), defined as nonsusceptibility of microorganisms, such as bacteria or fungi, to antimicrobial drugs, is a serious worldwide threat to public health, leading to an increase in mortality rates, prolonged illness, and higher medical costs, among others [1]. Several mechanisms are contributing to MDR, including alterations in the cell membrane, which reduces uptake of drugs, overexpression of drug-targeting enzymes, enzymatic degradation of antimicrobials, and, mainly, overexpression of efflux pumps [1].

Efflux pumps are membrane structures present in every bacterium and can be grouped into six families according to their substrates, composition, energy source, and number of transmembrane spanning regions. Within these, the most relevant families are the resistant-nodulation-division (RND) family, particularly important in Gram-negative bacteria, and the major facilitator superfamily (MFS), with greater impact in Gram-positive bacteria [2].

Aside from transport of antibacterial drugs and other molecules outside the bacterial cell, efflux pumps display the ability to potentiate resistance and virulence mechanisms, specifically biofilm production and quorum-sensing (QS) processes. In fact, efflux pumps are involved in the transport of both extracellular polymeric substances and signaling molecules responsible for formation of biofilm and QS. Furthermore, they can also indirectly modulate biofilm-related genes through inducer molecules and can promote bacterial aggregation [3,4]. As such, inhibition of efflux pumps can block a multitude of processes and provides many advantages from a therapeutic point of view.

In past years, many natural products and their synthetic analogues have been studied as bacterial efflux pump inhibitors (EPI) [5,6,7,8,9,10,11]. Flavonoids are natural compounds that possess a wide range of biological activities, including antimicrobial properties [12,13,14]. Flavonoids were also found to reduce the minimum inhibitory concentration (MIC) in association with commercially available antibiotics through modulation of bacterial efflux pumps [5]. In fact, several flavonoids isolated from natural sources have been described as bacterial EPI for all families of efflux pumps [15,16,17,18,19,20,21]. Among flavonoids, some chalcones were also described as EPI, particularly against MFS efflux pumps [22]. Recently, as a result of the search of new antimicrobial adjuvants by our research group, some chalcones have demonstrated the ability to potentiate the antimicrobial effect of antibiotics, this feature being associated with efflux pumps inhibition. In these studies, the presence of methoxy groups in the chalcone scaffold exerted a positive influence on efflux pump inhibitory activity, as well as on biofilm inhibition [23,24].

Hybridization of chalcones with other pharmacophores through the 1,2,3-triazole ring has enabled obtaining compounds with interesting antimicrobial activities [25,26]. Aside from being used as a linker, the 1,2,3-triazole has been associated with a wide range of biological activities, particularly antibacterial, and associated with improvement in the pharmacokinetic profile and stability of oxidative/reductive conditions and enzymatic degradation of triazole-bearing compounds [27]. Thus, considering the antimicrobial activity of methoxy chalcones and the heterocyclic 1,2,3-triazole ring, a small library of chalcone–triazole hybrids was synthesized in this work to further evaluate the antimicrobial activity and potential regarding reversion of some mechanisms of antimicrobial resistance in bacteria, namely inhibition of efflux pumps and biofilm formation.

## 2. Results and Discussion

### 2.1. Synthesis and Structure Elucidation

**Figure 1:**
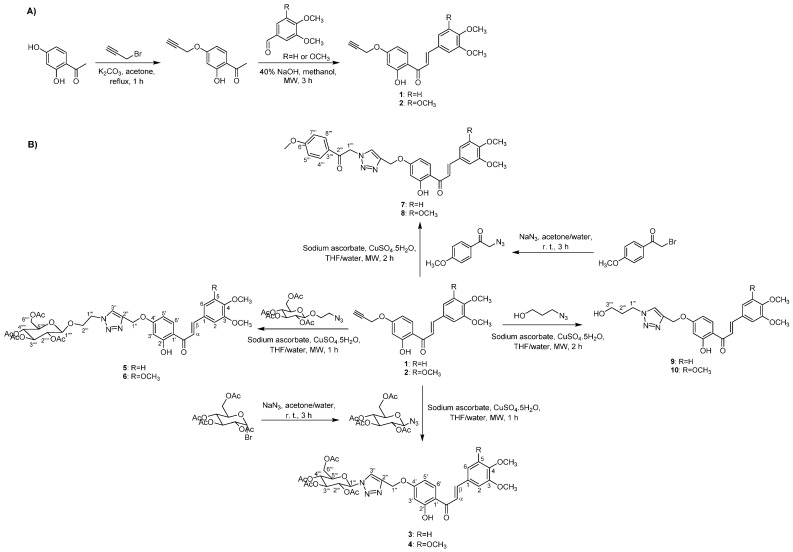
(**A**) Synthesis of intermediate chalcones **1**–**2**. (**B**) Synthesis of chalcone-1,2,3-triazole derivatives **3**–**10** by CuAAC reaction.

A series of chalcone hybrids bearing a 1,2,3-triazole moiety were synthesized according to the strategy illustrated in Figure 1. Synthesis of intermediate chalcones **1** and **2**, bearing a terminal alkyne, was accomplished by Claisen Schmidt condensation from appropriate acetophenone and benzaldehydes, as described by Pereira et al. (Figure 1A) [28]. Chalcone hybrids **3**–**6**, previously reported by our research group [28], and new chalcone derivatives **7**–**10** were prepared by copper(I)-catalyzed azide alkyne cycloaddition (CuAAC) from previously obtained chalcones **1**–**2** and different azides in the presence of copper sulfate pentahydrate and sodium ascorbate under microwave (MW) irradiation, affording **3**–**10** with moderate yields (Figure 1B). For preparation of chalcones **5**–**6** and **7**–**8**, the corresponding azides were prepared from acetobromo-α-d-glucose and 4-methoxyphenacyl bromide, respectively, in the presence of sodium azide in acetone/water at room temperature, which afforded azides in good yields. For synthesis of chalcone hybrids **9**–**10**, the azide was commercially available (Figure 1B).

New synthetic compounds **7**–**10** were characterized by ^1^H and ^13^C NMR and HRMS, and ^13^C NMR assignments were determined by 2D heteronuclear single quantum correlation (HSQC) and heteronuclear multiple bond correlation (HMBC) experiments. The NMR data of compounds **3**–**6** were in accordance with those previously reported [28]. The (*E*)-configuration of newly synthesized chalcones was confirmed by the coupling constants of the vinylic system (J_Hα-Hβ_ = 15.3–15.4 Hz). Moreover, the presence of characteristic signals of the triazole (δH-3′’: 8.27–8.24 s, δC2′’: 141.9–141.8 and δC3′’: 126.7–124.9) confirmed the formation of this heterocyclic ring.

### 2.2. Antimicrobial Activity and Potentiation of Antimicrobial Activity

Triazolyl chalcones, as well as their intermediates **1** and **2**, were tested for their potential to be used as antimicrobials. In this scope, four reference strains of bacteria included in the World Health Organization priority list for research and development of new antibiotics were used: *Escherichia coli* ATCC 25922, *Staphylococcus aureus* ATCC 25923, *Pseudomonas aeruginosa* ATCC 27853, and *Enterococcus faecalis* ATCC 29212 [29]. Moreover, the antifungal activity was evaluated against fungal species *Candida albicans*, *Aspergillus fumigatus*, and *Trichophyton rubrum*.

Compound **7** proved to be the only active compound, displaying an MIC of 32 µg/mL for the vancomycin-resistant *E. faecalis* strain tested. None of the compounds showed activity against the fungal strains, with MICs higher than the maximum tested concentration (128 µg/mL).

Chalcone derivatives were also tested for their potential to potentiate the activity of antibiotics in resistant bacterial strains. The models chosen were extended-spectrum β-lactamase-producing strain *Escherichia coli* SA/2 and vancomycin-resistant *Enterococcus faecalis* B3/101, two clinically relevant isolates [30]. Herein, a sub-MIC fixed concentration of the compounds was combined with serial dilutions of the antibiotic in order to observe the potential synergy between them. The results obtained can be viewed in Table 1.

It can be noted that the studied chalcones have moderate potentiating effects, which are more notable in the Gram-positive strain bacteria *E. faecalis* B3/101. In fact, while two compounds (**4** and **5**) were able to decrease the MIC of cefotaxime in *E. coli* SA/2 by two-fold, three compounds (**1**, **2**, and **5**) could do the same in *E. faecalis* B3/101, and chalcones **7** and **9** could even decrease the MIC of vancomycin by eight-fold and four-fold, respectively.

Afterwards, the potency of compounds **1**, **2**, **4**, **5**, **7**, and **9** was evaluated by the checkerboard assay, where the concentrations of the antibiotics and compounds were varying (CTX: 512—32 µg/mL; VAN: 1024—64 µg/mL; compounds: 64—1 µg/mL). The results obtained herein did not show any other effective concentrations of compounds than 64 µg/mL, and higher doses could not be tested as the percentage of DMSO would surpass the recommended 1% *v/v*.

While these results may suggest the potential of chalcones to restore the activity of antibiotics, further studies should be conducted in order to elucidate the exact mechanism through which this is happening, as well as synthesis of derivatives with a broader spectrum or a greater impact on MIC reduction.

### 2.3. Efflux Pump Inhibition Assay

The compounds were assessed for their capability of modulating EB accumulation on the methicillin- and oxacillin-resistant *Staphylococcus aureus* 272123 and *acrA* gene deleted *Salmonella enterica* serovar Typhimurium SL1344 strains by the automated EB method.

The relative fluorescence index (RFI) was calculated based on the means of relative fluorescence units, as can be seen in Table 2. The compounds were studied at 50 µM as the MICs for these strains were >100 µM (results not shown). Reserpine and CCCP were used at 25 µM as Gram-positive and Gram-negative reference EPIs, respectively, being the positive controls in this assay.

The results obtained from the real-time EB accumulation assay suggest that the compounds are more active towards the Gram-negative strain. In fact, none of the tested chalcone derivatives showed efflux pump inhibition in the *S. aureus* strain used in this assay. In contrast, five compounds (**3**, **5**–**8**) were able to increase the levels of EB inside the bacteria, which led to an increase in fluorescence. This is unlikely to be originated by the fluorescence of the compounds, as the results obtained do not suggest that the compounds presented fluorescent properties (Figures S1–S5, Supplementary Materials). In fact, some tested compounds presented negative RFI values, which means the fluorescence was lower than that of the negative control (DMSO) and, therefore, considered inactive.

Considering these promising results, docking studies were performed with the crystal structure of the AcrB (4DX5), AcrA (2F1M), and TolC (1EK9) portions of the AcrAB-TolC efflux system. For AcrB and AcrA, these studies were performed in two different sites: the substrate-binding site (SBS) and the hydrophobic trap (HT) for AcrB, and the helical hairpin (HH) and the lipoyl domain (LD) for AcrA [31]. For TolC, the region containing the lysine residues that interact with the 3,3′-dithiobis (sulfosuccinimidyl proprionate) (DTSSP) bifunctional crosslinker were considered as these residues are responsible for binding TolC to AcrA and the peptidoglycan [31] in order to further investigate the possible binding site of these compounds in the AcrAB-TolC efflux pump. As the substrate binding site (SBS) of the AcrB portion (PDB: 4DX5) [32] presented the best overall docking scores (Table S1, Supplementary Materials), it was chosen for molecular visualization with the five compounds that presented activity using the program PyMol.

The docking results showed that the compounds with the best scores for the SBS are **4**, **5**, and **7**, while **6**, **8**, and **9** are predicted to have a better affinity towards the HT. Experimentally, **6**, **7**, and **8** displayed favorable results regarding inhibition of Gram-negative efflux pumps, corroborating these results.

**Figure 2:**
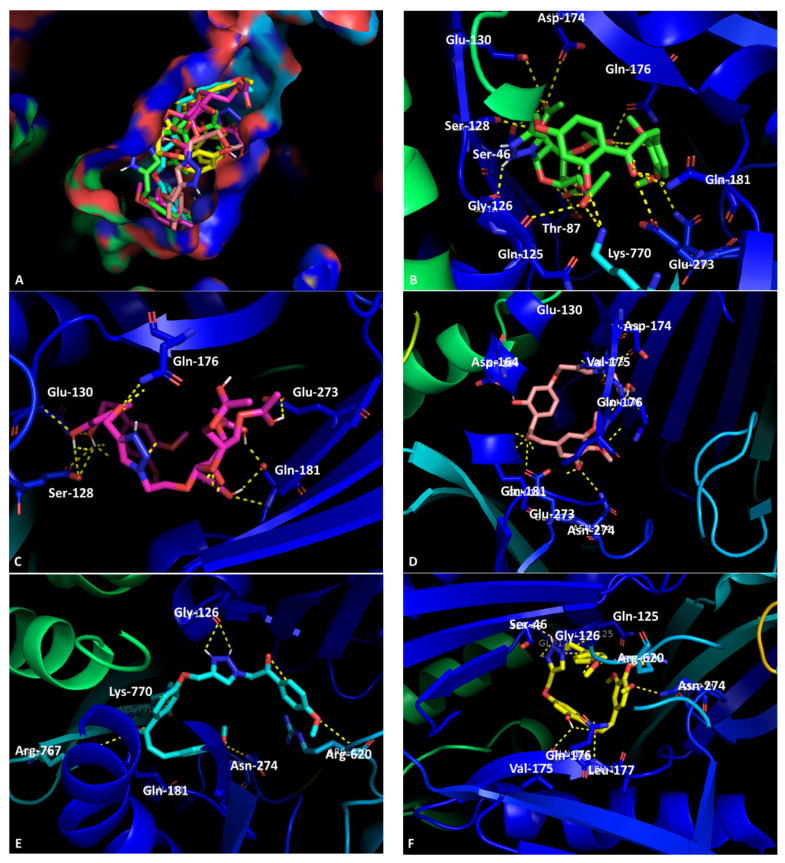
Molecular visualization of chalcones in the SBS of the AcrB portion of the AcrAB-TolC efflux system. (**A**) General view of compounds in the SBS (surface view); (**B**) interaction between compound **3** (green) and the SBS; (**C**) interaction between compound **5** (pink) and the SBS; (**D**) interaction between compound **6** (magenta) and the SBS; (**E**) interaction between compound **7** (blue) and the SBS; (**F**) interaction between compound **8** (yellow) and the SBS.

Upon initial analysis, all the compounds studied herein bind approximately in the same site of the SBS. Moreover, they are predicted to enter the pocket, as evidenced in Figure 2A. Closer examination of the interactions between the compounds and the target were made. First, compound **3** showed predicted interactions between Ser-46, Thr-87, Gln-176, and Asn-274 through polar interactions between carbonyls and hydroxyls present in both the compound and the efflux pump. Other polar interactions observed were with Gln-125, Gly-126, Ser-128, Glu-130, Asp-174, and Glu-237, while hydrogen bonds were observed between the carbonyl in the chalcone scaffold of compound **3** and Gln-181 and the C-2 hydroxyl and a carbonyl in the sugar moiety and Lys-770, as depicted in Figure 2B.

Chalcone derivative **5** established hydrogen bonds with Ser-128 through a carbonyl in the sugar moiety and an NH in the residue, as well as polar interactions. The residue Glu-130 was also involved in a polar interaction with an acetyl in the sugar moiety, while the NH_2_ in Gln-176 was predicted to interact with the oxygen bound to C-4′. Last, the hydroxyl in C-2′ was able to establish a hydrogen bond with both Glu-181 and Gln-273, while the chalcone carbonyl established a polar interaction and a hydrogen bond with the same residues, respectively (Figure 2C). Concerning compound **6**, it was predicted to interact with Asp-164 through a hydrogen bond between a hydroxyl in ring A and the carbonyl group of the residue, Asp-174 and Val-175, whose carbonyls establish hydrogen bonds with the nitrogens in the triazole ring, Gln-176, Leu-177, and Gln-181, with a polar interaction being established between the carbonyl and an oxygen in the compound, and Asn-274, which can establish a hydrogen interaction between the nitrogen in the residue and an oxygen in the methoxy groups present in ring B (Figure 2D).

Compound **7** is predicted to interact with Gly-126 through a hydrogen bond established via the triazole group. The carbonyl at position C-1 can establish hydrogen bonds with Gln-181, Lys-770, and Arg-767, while the oxygen at the methoxy group in ring B can establish a hydrogen bond with Asn-274. In terms of polar interaction, one can be found between the methoxy in the substituent ring and Arg-620 (Figure 2E). Last, the triazole group in compound **8** is predicted to establish a hydrogen interaction with Ser-46, Gln-125, and Gly-126. The hydroxyl in ring A can also form a hydrogen interaction with Val-175, and the carbonyl at C-1 with Gln-176 and Leu-177. The two last hydrogen interactions predicted are between the methoxy groups in the B ring and Asn-274 and Arg-620 (Figure 2F).

Based on these observations, both methoxy and triazole moieties were found to be important in the stabilization of the complex.

### 2.4. Inhibition of Biofilm Formation and Quorum-Sensing

Even though none of the compounds displayed efflux pump inhibition in the ethidium bromide accumulation assay, several compounds could interfere with the formation of biofilm in a different manner. As such, compounds **1**–**10** were tested for their potential to inhibit formation of biofilm in *S. aureus* ATCC 25923 and the methicillin- and oxacillin-resistant *S. aureus* 272123.

Thus, a biofilm formation inhibition assay was performed, and the results were presented as percentage (%) of biofilm formation inhibition. Reserpine was used as a positive control as it is not only an EPI but has also been described as an inhibitor of biofilm formation [33,34]. Thioridazine was also used as a positive control for *S. aureus* ATCC 25923. The results are presented in Table 3.

From the analysis of the results, it can be inferred that several compounds are active against formation of biofilm in the resistant strain, displaying underwhelming results in the ATCC strain. Considering the results obtained with *S. aureus* 272123, four compounds (**1**, **3**, **7,** and **10**) displayed greater effects than reserpine, and it can be hypothesized that the mechanism of action is different between the chalcone derivatives and reserpine as the compounds displayed no EPI activity. However, further studies would be necessary to confirm this hypothesis.

Bacterial communication or QS can contribute to resistance and virulence, for example, by promoting biofilm formation. Therefore, the potential of the chalcone derivatives to inhibit quorum-sensing was also studied. The strains used were *Serratia marcescens* AS-1 and *Chromobacterium violaceum* wt85 (wt85), which were inoculated as a single line. The other model used was *C. violaceum* CV026 (CV026), a Tn5 transposase-mutant, acyl-homoserine-lactone (AHL)-signal molecule indicator strain (produces purple violacein pigment in the presence of AHL), which is incapable of endogenous QS-signal molecule production but useful in detection of external stimuli, which was inoculated in a parallel line next to *Sphingomonas paucimobilis* Ezf 10-17 (EZF), an AHL-producing strain [35]. Moreover, bacteria of the *Chromobacterium* genus have been described to possess RND efflux systems [36].

The results obtained showed that the chalcones were not able to cause discoloration, and, therefore, no compounds described herein were able to inhibit quorum-sensing.

### 2.5. In Silico Prediction of Druglikeness and Toxicity

Considering that incorporation of the 1,2,3-triazole ring in the chalcone scaffold can improve the pharmacokinetic profile, the lipophilicity of the final compounds **3**–**10**, as well as the intermediates **1**–**2,** was predicted using SwissADME web server (http://www.swissadme.ch/, accessed on 6 November 2022) [37]. As shown in Table S2 (Supplementary Materials), all the compounds showed a Log P value according to the values preconized by the drug-likeness guidelines (0 < Log P < 5) and, excepting hybrids **7** and **8**, the hybridization improved the Log P profile. In order to predict the druglikeness, the compounds were screened for five rules of Medicinal Chemistry, namely Lipinski, Ghose, Veber, Egan, and Muegge (Figure S6, Supplementary Materials), using the same web server. Among them, hybrids **9** and **10**, as well as the intermediate chalcones, showed the highest compliance.

Chalcones **1**–**10** were also screened for rat oral acute toxicity using the ADMETlab 2.0 web server (https://admetmesh.scbdd.com/, accessed on 6 November 2022). Hybrids **3**–**10** displayed low toxicity (Figure S7, Supplementary Materials). The cytotoxicity of chalcone derivative **3** hit compound in EP and biofilm inhibitory assays was tested in mouse embryonic fibroblast cell lines (NIH/3T3) using the 3-(4,5-dimethylthiazol-2-yl)-2,5-diphenyltetrazolium bromide (MTT) assay. The IC_50_ of the tested compound was found to be 80.07 ± 3.59 µM, higher than the range of concentrations investigated in the resistance mechanisms.

## 3. Materials and Methods

### 3.1. Chemistry

#### 3.1.1. Synthesis and Structure Elucidation of Chalcones

MW reactions were performed using a glassware setup for atmospheric pressure reactions and a 100 mL Teflon reactor (internal reaction temperature measurements with a fiber-optic probe sensor) and were carried out using an Ethos MicroSYNTH 1600 Microwave Labstation from Milestone (Bergamo, Italy). The reactions were monitored by analytical thin-layer chromatography (TLC). Purifications of compounds were carried out by flash chromatography using Macherey-Nagel silica gel 60 (0.04–0.063 mm) (Macherey-Nagel, Düren, Germany), preparative TLC using Macherey-Nagel silica gel 60 (GF_254_) plates (Macherey-Nagel, Düren, Germany) and crystallization. Melting points were obtained in a Köfler microscope and are uncorrected. ^1^H and ^13^C NMR spectra were taken in CDCl_3_ or DMSO-d_6_ at room temperature on Bruker Avance 300 and 500 instruments (300 MHz or 500 MHz for ^1^H and 75 or 120 MHz for ^13^C, Bruker Biosciences Corporation, Billerica, MA, USA). Chemical shifts are expressed in δ (ppm) values relative to tetramethylsilane (TMS) as an internal reference; ^13^C NMR assignments were made by 2D (HSQC and HMBC) NMR experiments (long-range ^13^C–^1^H coupling constants were optimized to 7 Hz). HRMS mass spectra of compounds **7** and **9** were performed on an APEXQe FT-ICR MS (Bruker Daltonics, Billerica, MA, USA), equipped with a 7T actively shielded magnet, at C.A.C.T.I.-University of Vigo, Spain. Ions were generated using a Combi MALDI-electrospray ionization (ESI) source. HRMS spectra of compounds **8** and **10** were performed on an LTQ OrbitrapTM XL hybrid mass spectrometer (Thermo Fischer Scientific, Bremen, Germany) controlled by *LTQ Tune Plus 2.5.5* and *Xcalibur 2.1.0.* at CEMUP—University of Porto, Portugal. Intermediate chalcones **1**–**2** and triazolyl glycosylated chalcones **3**–**6** were synthesized and characterized according to the method described previously by our research group [28]. The new chalcone derivatives **7**–**10** were synthesized and characterized as follows.

##### Synthesis of Chalcones **7** and **8**

Chalcones **7** and **8** were prepared from propargylated chalcones **1** and **2**, respectively, and 4-methoxyphenacyl azide. For preparation of 4-methoxyphenacyl azide, 4-methoxyphenacyl bromide (0.200 g; 0.873 mmol) was dissolved in 6 mL of acetone and a solution of sodium azide (0.0709 g, 1.09 mmol) in 3 mL of water was added. The reaction was performed at room temperature for 3 h until the end of reaction. Then, acetone was evaporated under reduced pressure and the product insolubilized from water. The solid was filtered and dried to afford 4-methoxyphenacyl azide with 66% yield. NMR data of 4-methoxyphenacyl azide were in accordance with previously reported [38].

For preparation of chalcones **7** and **8**, a solution of **1** (0.296 mmol) or **2** (0.678 mmol) and 4-methoxyphenacyl azide (0.325 mmol or 1.357 mmol) in THF/water solvent mixture (2:1; 30 mL), sodium ascorbate (1.18 mmol or 2.71 mmol), and copper (II) sulfate pentahydrate (0.591 mmol or 1.357 mmol) were added. The reaction mixture was heated under MW irradiation of 250 W for 2 h at 70 °C. After cooling, the reaction mixture was filtered and the THF was evaporated under reduced pressure and the water suspension was extracted with ethyl acetate (3 × 20 mL). The combined organic layers were washed with water (2 × 20 mL), dried with anhydrous sodium sulfate, evaporated under reduced pressure, and then purified by flash column chromatography (SiO_2_; *n*-hexane: ethyl acetate, 5:5) (**7**) or crystallized from chloroform (**8**).

(*E*)-3-(3,4-dimethoxyphenyl)-1-(2-hydroxy-4-((1-(2-(4-methoxyphenyl)-2-oxoethyl)-1*H*-1,2,3-triazol-4-yl)methoxy)phenyl)prop-2-en-1-one (**7**). Yellow solid; yield: 64%; m.p.: 90–92 °C (ethyl acetate); ^1^H NMR (500 MHz, DMSO), δ: 13.67 (1H, s, OH-2′), 8.34 (1H, d, J = 9.1 Hz, H-6′), 8.24 (1H, s, H-3″), 8.06 (2H, d, J = 8.9 Hz, H-4‴ and H-8‴), 7.91 (1H, d, J = 15.3 Hz, H-α), 7.81 (1H, d, J = 15.3 Hz, H-β), 7.59 (1H, d, J = 2.0 Hz, H-2), 7.42 (1H, dd, J = 8.4, 2.0 Hz, H-6), 7.13 (2H, d, J = 8.9 Hz, H-5‴ and H-7‴), 7.04 (1H, d, J = 8.4 Hz, H-5), 6.72 (1H, d, J = 2.5 Hz, H-3′), 6.68 (1H, dd, J = 9.0, 2.5 Hz, H-5′), 6.16 (2H, s, H-1‴), 5.32 (2H, s, H-1″), 3.88 (3H, s, 6‴-OCH_3_), 3.87 (3H, s, 3-OCH_3_), 3.83 (3H, s, 4-OCH_3_). ^13^C NMR (126 MHz, DMSO) δ: 192.0 (CO), 190.4 (C2‴), 165.8 (C2′), 164.6 (C4′), 164.0 (C6‴), 151.6 (C4), 149.1 (C3), 145.0 (Cβ), 141.8 (C2″), 132.7 (C6′), 130.6 (C4‴ and C8‴), 127.4 (C1), 127.0 (C3‴), 126.7 (C3″), 124.6 (C6), 118.4 (Cα), 114.3 (C5‴ and C7‴), 114.0 (C1′), 111.5 (C5), 110.8 (C2), 107.8 (C5′), 101.8 (C3′), 61.6 (C1″), 55.8, 55.7, 55.6, 55.6 (C1‴, 6‴-OCH_3_, 3-OCH_3_ and 4-OCH_3_). HRMS (ESI^+^) m/z calcd for C_29_H_28_N_3_O_7_ [M + H^+^] 530.19218, found 530.19252.

(*E*)-1-(2-hydroxy-4-((1-(2-(4-methoxyphenyl)-2-oxoethyl)-1*H*-1,2,3-triazol-4-yl)methoxy)phenyl)-3-(3,4,5-trimethoxyphenyl)prop-2-en-1-one (**8**). Yellow solid; yield: 70%; m.p.: 103–105 °C (chloroform); ^1^H NMR (300 MHz, DMSO), δ: 13.58 (1H, s, OH-2′), 8.35 (1H, d, J = 8.9 Hz, H-6′), 8.24 (1H, s, H-3″), 8.06 (2H, d, J = 8.4 Hz, H-4‴ and H-8‴), 7.97 (1H, d, J = 15.4 Hz, H-α), 7.80 (1H, d, J = 15.4 Hz, H-β), 7.27 (2H, s, H-2 and H-6), 7.13 (2H, d, J = 8.4 Hz, H-5‴ and H-7‴), 6.74 (1H, d, J = 2.6 Hz, H-3′), 6.69 (1H, dd, J = 9.0, 2.3 Hz, H-5′), 6.16 (2H, s, H-1‴), 5.33 (2H, s, H-1″), 3.87 (9H, s, 3-OCH_3_, 5-OCH_3_, 6‴-OCH_3_), 3.72 (3H, s, 4-OCH_3_). ^13^C NMR (75 MHz, DMSO) δ: 192.0 (CO), 190.4 (C2‴), 165.8 (C2′), 164.8 (C4′), 163.9 (C6‴), 153.1 (C3 and C5), 144.8 (Cβ), 141.8 (C2″), 140.0 (C4), 132.8 (C6′), 130.6 (C4‴ and C8‴), 130.1 (C1), 127.0 (C3‴), 126.7 (C3″), 120.2 (Cα), 114.2 (C5‴ and C7‴), 114.0 (C1′), 107.9 (C5′), 106.8 (C2 and C6), 101.8 (C3′), 61.6 (C1″), 60.2 (4-OCH_3_), 56.2 (3-OCH_3_ and 5-OCH_3_), 55.7 (6‴-OCH_3_), 55.6 (C1‴). HRMS (ESI^+^) m/z calcd for C_30_H_30_N_3_O_8_ [M + H^+^] 560.20274, found 560.20163.

##### Synthesis of Chalcones **9** and **10**

To a solution of **1** (0.296 mmol) or **2** (0.678 mmol) and 3-azido-1-propanol (0.325 mmol or 1.018 mmol) in THF/water solvent mixture (2:1; 30 mL), sodium ascorbate (1.18 mmol or 2.71 mmol) and copper(II) sulfate pentahydrate (0.591 mmol or 1.357 mmol) were added. The reaction mixture was heated under MW irradiation of 250 W for 2 h at 70 °C. After cooling, the reaction mixture was filtered and THF was evaporated under reduced pressure. Then, the water suspension was extracted with ethyl acetate (3 × 20 mL). The combined organic layers were washed with water (2 × 20 mL), dried with anhydrous sodium sulfate, and evaporated under reduced pressure, and then purified by crystallization from methanol (**9**) or chloroform (**10**).

(*E*)-3-(3,4-dimethoxyphenyl)-1-(2-hydroxy-4-((1-(3-hydroxypropyl)-1*H*-1,2,3-triazol-4-yl)methoxy)phenyl)prop-2-en-1-one (**9**). Yellow solid; yield: 53%; m.p.: 135–137 °C (methanol); ^1^H NMR (300 MHz, DMSO), δ: 13.65 (1H, s, OH-2′), 8.32 (1H, d, J = 9.1 Hz, H-6′), 8.27 (1H, s, H-3″), 7.90 (1H, d, J = 15.3 Hz, H-α), 7.80 (1H, d, J = 15.3 Hz, H-β), 7.58 (1H, d, J = 2.0 Hz, H-2), 7.42 (1H, dd, J = 8.4, 2.0 Hz, H-6), 7.03 (1H, d, J = 8.4 Hz, H-5), 6.68 (1H, d, J = 2.5 Hz, H-3′), 6.64 (1H, dd, J = 8.9, 2.5 Hz, H-5′), 5.25 (2H, s, H-1″), 4.70 (1H, t, J = 5.0 Hz, OH-3‴), 4.44 (2H, t, J = 7.1 Hz, H-1‴), 3.87 (3H, s, 3-OCH_3_), 3.82 (3H, s, 4-OCH_3_), 3.43-3.40 (2H, m, H-3‴), 2.01-1.92 (2H, m, H-2‴). ^13^C NMR (75 MHz, DMSO) δ: 192.0 (CO), 165.8 (C2′), 164.6 (C4′), 151.6 (C4), 149.1 (C3), 145.0 (Cβ), 141.9 (C2″), 132.7 (C6′), 127.4 (C1), 124.9 (C3″), 124.6 (C6), 118.4 (Cα), 114.0 (C1′), 111.6 (C5), 110.8 (C2), 107.8 (C5′), 101.8 (C3′), 61.6 (C1″), 57.5 (C3‴), 55.8 (3-OCH_3_), 55.7 (4-OCH_3_), 46.8 (C1‴), 33.0 (C2‴). HRMS (ESI^+^) m/z calcd for C_23_H_26_N_3_O_6_ [M + H^+^] 440.18161, found 440.18172.

(*E*)-1-(2-hydroxy-4-((1-(3-hydroxypropyl)-1*H*-1,2,3-triazol-4-yl)methoxy)phenyl)-3-(3,4,5-trimethoxyphenyl)prop-2-en-1-one (**10**). Yellow solid; yield: 55%; m.p.: 160–162 °C (chloroform); ^1^H NMR (300 MHz, DMSO), δ: 13.56 (1H, s, OH-2′), 8.33 (1H, d, J = 9.1 Hz, H-6′), 8.27 (1H, s, H-3″), 7.96 (1H, d, J = 15.4 Hz, H-α), 7.80 (1H, d, J = 15.4 Hz, H-β), 7.27 (2H, s, H-2 and H-6), 6.70 (1H, d, J = 2.4 Hz, H-3′), 6.66 (1H, dd, J = 8.9, 2.5 Hz, H-5′), 5.26 (2H, s, H-1″), 4.68 (1H, t, J = 5.0 Hz, OH-3‴), 4.44 (2H, t, J = 7.1 Hz, H-1‴), 3.87 (6H, s, 3-OCH_3_ and 5-OCH_3_), 3.72 (3H, s, 4-OCH_3_), 3.43-3.38 (2H, m, H-3‴), 2.01-1.92 (2H, m, H-2‴). ^13^C NMR (75 MHz, DMSO) δ: 192.0 (CO), 165.8 (C2′), 164.7 (C4′), 153.1 (C3 and C5), 144.9 (Cβ), 141.8 (C2″), 140.0 (C4), 132.8 (C6′), 130.1 (C1), 124.9 (C3″), 120.2 (Cα), 114.0 (C1′), 107.9 (C5′), 106.8 (C2 and C6), 101.8 (C3′), 61.6 (C1″), 60.2 (4-OCH_3_), 57.4 (C3‴), 56.2 (3-OCH_3_ and 5-OCH_3_), 46.7 (C1‴), 32.9 (C2‴). HRMS (ESI^+^) m/z calcd for C_24_H_28_N_3_O_7_ [M + H^+^] 470.19218, found 470.19027.

### 3.2. Docking Studies

The crystal structures of the AcrB (PDB: 4DX5) [39], AcrA (PDB: 2F1M) [40], and TolC (PDB: 1EK9) [41] portions of the AcrAB-TolC bacterial efflux system, downloaded from the protein databank (PDB) [42], were used for this study. The NorA efflux pump does not have an available crystal structure, and a homology model was prepared. The model was generated using the Swiss Model server [43] and the sequence deposited in Uniprot (Q5HHX4) [44] using the EmrD pump from *E. coli* (PDB: 2GFP) as the homolog, as described in [45]. The sequence similarity was 0.28, the coverage was 0.91, and the sequence identity 17.33%. The known AcrAB-TolC inhibitors minocycline and phenyl-arginyl-β-naphthylamide, along with the tested compounds, were drawn with ChemDraw (PerkinElmer Informatics) and minimized using ArgusLab. Docking was carried out using AutoDock Vina (Scripps, CA, USA) using a stochastic genetic algorithm. The methodology used was flexible ligand and rigid receptor [46]. The search exhaustiveness was 8, and the grid size varied according to the site being analyzed and in order to fit the sites described in [31,32]. The position and dimensions of the sites are in Table 4. The top nine poses were collected for each molecule, and the lowest docking score value was associated with the most favorable binding conformation.

### 3.3. Biological Activity

#### 3.3.1. Culture Media and Chemicals

The culture media used in the experiments: cation-adjusted Mueller–Hinton broth (MHB II) was purchased from Sigma-Aldrich, St. Louis, MO, USA and Biokar Diagnostics, Allone, Beauvais, France, the Luria-Bertani broth (LB-B) from Sigma, St. Louis, MO, USA, Tryptic Soy broth (TSB) was bought from Scharlau Chemie S.A., Barcelona, Spain Tryptic-Soy agar (TSA) was purchased from Biokar Diagnostics, Allone, Beauvais, France), Sabouraud Dextrose Agar (SDA) from Bio-Mérieux, Marcy L’Etoile, France, RPMI-1640 broth medium from Biochrom AG, Berlin, Germany, which was buffered with 3-(*N*-morpholino) propanesulfonic acid (MOPS), purchased from Sigma-Aldrich, St. Louis, MO, USA, to pH 7.0. The modified Luria–Bertani agar (LB*-A) was prepared in-house according to the formula 1.0 g yeast extract (Merck, Darmstadt, Germany), 10.0 g tryptone (Biolab, Budapest, Hungary), 10.0 g NaCl (Molar Chemicals, Halásztelek, Hungary), 1.0 g K_2_HPO_4_ (Biolab, Budapest, Hungary), 0.3 g MgSO_4_ × 7H_2_O (Reanal, Budapest, Hungary), 5 mL Fe-EDTA stock solution, and 20.0 g of bacteriological agar (Molar Chemicals, Halásztelek, Hungary) per 1 L of media.

Dimethyl sulfoxide (DMSO), MTT, sodium dodecyl sulfate (SDS), phosphate-buffered saline (PBS; pH 7.4), ethidium bromide (EB), reserpine, thioridazine, carbonyl cyanide 3-chlorophenylhydrazone (CCCP), promethazine (PMZ), and crystal violet (CV) were purchased from Sigma-Aldrich Chemie GmbH (Steinheim, Germany). The antibiotic cefotaxime (CTX) was purchased from Duchefa Biochemie (Haarlem, The Netherlands).

#### 3.3.2. Bacterial and Fungal Strains

As Gram-positive strains, *Staphylococcus aureus* American Type Culture Collection (ATCC) 25923, *Enterococcus faecalis* ATCC 29212, methicillin and ofloxacin-resistant *Staphylococcus aureus* 272123 clinical isolate [47], and vancomycin-resistant enterococci (VRE) *E. faecalis* B3/101 [30] were used. As Gram-negative strains, *Escherichia coli* ATCC 25922*, Pseudomonas aeruginosa* ATCC 27853, *acrA* gene inactivated mutant *Salmonella enterica* serovar Typhimurium SL1344, and clinical isolates of the extended-spectrum β-lactamase producer (ESBL) *E. coli* SA/2 were investigated in this study.

For the QS tests, all the strains used were Gram-negative. The bacteria used were *Chromobacterium violaceum* wild type 85 (wt85) characterized by the acyl-homoserine-lactone (AHL) signal-molecule-mediated production of the purple violacein pigment, capable of endogenous QS signal molecule production (*N*-hexanoyl-l-HSL), *C. violaceum* CV026 (CV026), a Tn5 transposase-mutant, AHL-signal molecule indicator strain (produces purple violacein pigment in the presence of AHL), which is incapable of endogenous QS-signal molecule-production but useful in the detection of external stimuli, *Sphingomonas paucimobilis* Ezf 10-17 (EZF), AHL-producing-strain (used with *C. violaceum* CV026), and *Serratia marcescens* AS-1, characterized by AHL signal-molecule-mediated production of the orange–red pigment prodigiosin (2-methyl-3-pentyl-6-methoxyprodigiosin), capable of endogenous QS signal molecule production (*N*-hexanoyl-l-HSL), were applied [35].

As yeast, *Candida albicans* ATCC 10231, and, as filamentous fungi, *Aspergillus fumigatus* ATCC 204305 and a dermatophyte (*Trichophyton rubrum* FF5, clinical isolate) were used. Cultures were obtained in SDA.

#### 3.3.3. Antibacterial Assay

The antibacterial activity was assessed by determining the MIC of the compounds. It was evaluated by microdilution method in a 96-well plate according to the Clinical and Laboratory Standard Institute (CLSI) guidelines [48]. The media applied was MHB II. The concentrations tested ranged from 64 µg/mL to 4 µg/mL. The MIC was determined by visual inspection. DMSO was used as a solvent for the compounds in subinhibitory concentrations (1 % (*v*/*v*)).

#### 3.3.4. Antifungal Assay

The susceptibility tests for yeasts were performed based on the CLSI description for the broth microdilution method on the reference document M27A-3 for yeasts [49] and M38-A2 [50] for filamentous fungi in RPMI-1640 broth culture media. The concentrations tested ranged from 128 µg/mL to 32 µg/mL. The MIC was considered the lowest concentration that was able to totally inhibit growth when compared to control (non-treated microorganism in culture medium). A DMSO control (microorganism in RPMI with DMSO (1% (*v*/*v*)) was included.

#### 3.3.5. Combination with Antibiotics

The combined effect of the compounds and clinically relevant antimicrobial drugs was evaluated by determining the MIC of the antibiotic in the presence of each compound. Briefly, when it was not possible to determine an MIC value for the test compound, the MIC values of CTX and VAN for *E. coli* SA/2 and *E. faecalis* B3/101, respectively, were evaluated in the presence of the highest concentration of each compound tested in previous assays. The antibiotic tested was serially diluted, whereas the concentration of each compound was kept fixed. Antibiotic MICs were determined as described above.

Based on the results obtained, the checkerboard assay was performed in order to evaluate the effects of different concentrations of compounds **1**, **2**, **4**, **5**, **7**, and **9** along with the antibiotics CTX (512—32 µg/mL) and VAN (1024—64 µg/mL) against *E. coli* SA/2 and *E. faecalis* B3/101, respectively. Briefly, the stock solutions and serial twofold dilutions of each compound and antibiotic to at least double the MIC were prepared according to the recommendations of CLSI [51]. The compound to be tested was serially diluted along the ordinate, while the antibiotic was diluted along the abscissa. A bacterial inoculum equal to a 0.5 McFarland turbidity standard was prepared in MHB. Each microtiter plate well was inoculated with 100 µL of a bacterial inoculum of 5 × 10^5^ CFU/mL, and the plates were incubated overnight at 37 °C.

#### 3.3.6. Efflux Pump Inhibition Assays

The compounds were evaluated for their ability to inhibit efflux pumps in *S.* Typhimurium SL1344 and *S. aureus* 272123 by real-time fluorimetry, monitoring the intracellular accumulation of EB, an efflux pump substrate. This was determined by the automated method using a CLARIOstar Plus plate reader (BMG Labtech, Ortenberg, Germany). Reserpine and CCCP were applied at 25 µM as positive controls, and the solvent DMSO was applied at 1% (*v*/*v*). The bacterial strains were incubated in an appropriate culture media (TSB—*S. aureus* 272123; LB-B—*S.* Typhimurium SL1344) at 37 °C until they reached an optical density (OD) between 0.4 and 0.6 at 600 nm. The culture was centrifuged at 13,000× *g* for 3 min, and the pellet washed and resuspended with phosphate buffered saline (PBS, pH 7.4). The suspension was centrifuged again in the same conditions and resuspended in PBS.

The compounds were applied at one-third MIC concentration in a solution of a non-toxic concentration of EB (1 µg/mL) in PBS. If the compound presented an MIC > 100 µM, it would be tested at 50 µM. Then, 50 µL of this solution was transferred into a 96-well black microtiter plate (Greiner Bio-One Hungary Kft, Mosonmagyaróvár, Hungary), and 50 µL of bacterial suspension (OD_600_ 0.4–0.6) was added to each well. The plates were placed into the CLARIOstar plate reader, and the fluorescence was monitored at excitation and emission wavelengths of 530 nm and 600 nm, respectively, every minute for one hour on a real-time basis. From the real-time data, the activity of the compounds, namely the relative fluorescence index (RFI) of the last time point (minute 60) of the EB accumulation assay, was calculated according to the following formula:

(1) $$\mathrm{RFI}=\frac{{\mathrm{RF}}_{\mathrm{treated}}-{\mathrm{RF}}_{\mathrm{untreated}}}{{\mathrm{RF}}_{\mathrm{untreated}}}$$

where RF_treated_ is the relative fluorescence (RF) at the last time point of EB accumulation curve in the presence of the compound, and RF_untreated_ is the RF at the last time point of the EB accumulation curve of the untreated control, having only the solvent (DMSO) control [52]. The accumulation curves were designed using Microsoft Excel^®^. The samples were tested in triplicate, and the RFI presented comes from the average of these three values. The accumulation curves present the mean of the RF over the cycles performed. The standard deviation (SD) was calculated automatically and included in the RF curves.

#### 3.3.7. Inhibition of Biofilm Formation

The chalcone derivatives were tested for their ability to decrease the formation of biofilm. The strains used were the Gram-positive *S. aureus* ATCC 25923 and *S. aureus* 272123. Detection of the biofilm formation was possible with the use of the dye crystal violet (CV; 0.1% (*v*/*v*)). The initial inoculum was incubated in TSB overnight and then diluted to an OD_600_ of 0.1. Then, the bacterial suspension was added to 96-well microtiter plates and the compounds were added at half the MIC. If the MIC was >100 µM, the compound would be added at the concentration of 100 µM. The final volume in each well was 200 µL. Reserpine and thioridazine were used as positive control.

The plates were incubated at 30 °C for 48 h, with gentle stirring (100 rpm). After this incubation period, the TSB medium was discarded, and the plates were washed with tap water to remove unattached cells. Afterwards, 200 µL of a 0.1% (*v*/*v*) CV solution were added to the wells and incubated for 15 min at room temperature. Then, the CV solution was removed from the wells, and the plates were washed again with tap water, and 200 µL of a 70 % ethanolic solution were added to the wells.

The biofilm formation was determined by measuring the OD_600_ using a Multiscan EX ELISA plate reader (Thermo Labsystems, Cheshire, WA, USA). The anti-biofilm effect of the compounds was expressed in the percentage (%) of decrease of biofilm formation [53].

#### 3.3.8. Quorum-Sensing Assay

The QS inhibitory effect of the compounds was examined on the EZF and the sensor CV026 strains, on the wt85 strain and on *S. marcescens*. The method used was the parallel inoculation method, where pair combinations of the used sensor strain CV026 and the *N*-acyl-homoserine lactone (AHL) producing strain EZF were inoculated directly onto the LB *-A agar surface in parallel, at an approximate distance of 5 mm from each other. *S. marcescens* AS-1 and wt85 were inoculated as a single line. Filter paper disks (7 mm in diameter) were placed on the center of the inoculated line(s) and impregnated with 8 µL of a solution of 10 mM of the compounds. Promethazine (PMZ) was used as a positive control.

The agar plates were incubated at room temperature (20 °C) for 24–48 h. The QS inhibition was accessed visually by determining the inhibition of pigment production. The discolored, but intact, bacterial colonies were measured with a ruler [54,55].

#### 3.3.9. Cytotoxicity in Mouse Embryonic Fibroblasts

Compound **3** was tested in the same concentrations as the antibacterial activity assay and prepared from the same 10 mM stock solution in DMSO, diluted freshly on the day of the experiment in cell culture media.

The cell line used was mouse fibroblasts (NIH/3T3, ATCC CRL-1658TM), cultivated in DMEM (Gibco 52100-039) and supplemented with 10% heat-inactivated fetal bovine serum (Biowest, VWR International Kft, Debrecen, Hungary), 2 mM of L-glutamine, 1 mM Na pyruvate, 100 U/L and 10 mg/L of penicillin/streptomycin mixture (Sigma-Aldrich Chemie GmbH, Steinheim, Germany), respectively, and 0.1% nystatin (8.3 g/L in ethylene glycol). Using a combination of 0.25% Trypsin-Versene (EDTA) solution for 5 min at 37 °C, the adherent cells were detached. Prior to each cytotoxicity assay, the cells were seeded in untreated 96-well flat-bottom microtiter plates, which was followed by a four-hour incubation period in a humidified atmosphere (5% CO_2_, 95% air) at 37 °C [56].

The MTT assay was used to determine the cytotoxicity of the compounds against NIH/3T3 cells. Prior to the assay, the cells were seeded for four hours using 1 × 10^4^ cells/well. The compounds were added by 2-fold serial dilutions to the cells distributed into 96-well flat bottom microtiter plates starting with 100 μM. Then, after the plates had been incubated for 24 h, a solution of MTT in PBS was added to each well, followed by a four-hour incubation period. After this, 100 μL of SDS (10% in a 0.01 M HCl solution) was added to each well and incubated overnight at 37 °C. As a positive control, the drug doxorubicin was used. Cell growth was determined in quadruplicate by measuring OD at λ = 540 nm (reference 630 nm) in a Multiscan EX ELISA reader (Thermo Labsystems, Cheshire, WA, USA). The percentage of inhibition of cell growth was determined as follows:

(2) $$100-\left(\frac{{\mathrm{OD}}_{\mathrm{sample}}-{\mathrm{OD}}_{\mathrm{medium}\;\mathrm{control}}}{{\mathrm{OD}}_{\mathrm{cell}\;\mathrm{control}}-{\mathrm{OD}}_{\mathrm{medium}\;\mathrm{control}}}\right)\times 100$$

The results were expressed as the mean ± SD, and the IC_50_ values were obtained by best fitting the dose-dependent inhibition curves in GraphPad Prism 5.03 for Windows software.

## 4. Conclusions

In this study, a small library of chalcones was synthesized and tested for the potential to act as antimicrobials and/or adjuvants in multidrug-resistant bacteria. Although only compound **7** was able to act as antibacterial against *E. faecalis* (32 µg/mL), six compounds (**1**, **2**, **4**, **5**, **7,** and **9**) demonstrated to revert the resistance in *E. coli* SA/2 and/or *E. faecalis* B3/101 bacterial strains. Moreover, it should be noted that these compounds demonstrated to be more effective in reversion of resistance in Gram-positive bacterial strain *E. faecalis* B3/101. Among them, the 3,4-dimethoxychalcone bearing an aromatic ring linked to the triazole group (**7**) proved to be the most promising compound in potentiation of vancomycin activity against an *E. faecalis* strain resistant to this glycopeptide. Concerning efflux pump inhibitory activity, triazolyl chalcones **3** and **5**–**8** were successful in the inhibition when the efflux of ethidium bromide in the Gram-negative strain was used, which could mean they act on AcrB, TolC, or even in different efflux systems present in this model, which may highlight the importance of this moiety on the activity. Even though the AcrAB-TolC efflux system is of great importance to the efflux of compounds in Gram-negative bacteria, it is not the only one. Therefore, genetic studies could be performed in order to assess the bacterial response in the presence of the active compounds.

Compounds **1**, **3**, **6**–**8**, and **10** were also able to inhibit biofilm formation in methicillin- and oxacillin-resistant *S. aureus* 272123. Considering that the majority of the active compounds were hybrids of chalcones with glycosyl, aromatic, and propanol groups linked by the 1,2,3-triazole ring, the importance of this group of compounds in the search for effective antimicrobial agents can be highlighted. In fact, several triazolyl chalcone derivatives have shown antimicrobial activity; however, to the best of our knowledge, this is the first time in which the mechanisms underlying the resistance of resistant bacterial strains were studied for this group of compounds.

When analyzing the results of all the assays, it appears that compound **7** proved to be the most promising of this series of compounds as it showed activity in the highest number of tests performed. Further studies on this hit compound may elucidate the precise mechanism through which it inhibits the efflux of EB. On the other hand, new derivatives can be synthesized, with compound **7** as a template, to develop more broad-spectrum compounds with both EPI and antibiofilm properties.

## Figures and Tables

**Table 1:** Ability of chalcones **1–10** to potentiate the activity of antibiotics in resistant bacterial strains.

Compound	*E. coli* SA/2	*E. faecalis* B3/101
	MIC CTX = 256 µg/mL (562 µM)	MIC VAN = 1024 µg/mL (707 µM)
	MIC Reduction	
**1**	None	2-fold
**2**	None	2-fold
**3**	None	None
**4**	2-fold	None
**5**	2-fold	2-fold
**6**	None	None
**7**	None	8-fold
**8**	None	None
**9**	None	4-fold
**10**	None	None

CTX, cefotaxime; MIC, minimum inhibitory concentration; VAN, vancomycin.

**Table 2:** Relative fluorescence index of chalcone derivatives **1–10**.

Compound	RFI ± SD	
	*S. aureus* 272123	*S*. Typhimurium SL1344
**1**	−0.06 ± 0.11	0.01 ± 0.02
**2**	−0.04 ± 0.20	0.02 ± 0.03
**3**	0.06 ± 0.05	0.45 ± 0.02
**4**	−0.09 ± 0.05	0.15 ± 0.05
**5**	0.20 ± 0.08	0.40 ± 0.06
**6**	0.33 ± 0.04	0.52 ± 0.07
**7**	0.06 ± 0.11	0.85 ± 0.02
**8**	0.05 ± 0.02	0.59 ± 0.12
**9**	0.10 ± 0.09	−0.01 ± 0.01
**10**	0.17 ± 0.06	−0.06 ± 0.03
**Reserpine**	0.48 ± 0.04	-
**CCCP**	-	0.40 ± 0.06

CCCP—carbonyl cyanide *m*-chlorophenyl hydrazone.

**Table 3:** Percentage of biofilm inhibition of chalcones **1**–**10**.

Compound	Biofilm Inhibition (%) ± SD	
	*S. aureus* ATCC 25923	*S. aureus* 272123
**1**	0.21 ± 1.82	94.58 ± 1.15
**2**	41.10 ± 2.12	32.77 ± 3.65
**3**	0	85.76 ± 2.61
**4**	57.40 ± 1.64	61.23 ± 4.63
**5**	0	36.89 ± 1.37
**6**	1.29 ± 0.42	59.56 ± 2.51
**7**	0.55 ± 2.00	89.36 ± 0.56
**8**	0	72.33 ± 0.89
**9**	0	26.47 ± 0.29
**10**	0.08 ± 0.34	83.51 ± 1.02
**Thioridazine**	97.07 ± 1.01	ND
**Reserpine**	0.53 ± 1.05	81.11 ± 1.18

ND: not determined.

**Table 4:** Position and dimensions of the grid used to perform the docking studies.

Structure	Site	Position			Dimension		
		X	Y	Z	X	Y	Z
AcrA (2F1M)	HH	27.4205	14.1758	175.9638	15.9628	12.8742	17.6756
	LD	26.8634	−2.5985	207.5824	15.9628	11.6319	27.9448
AcrB (4DX5)	SBS	24.3266	−32.1670	−7.0000	18.4129	26.7613	20.1435
	HT	20.8792	17.7378	−7.0708	14.5855	17.7378	15.3042
TolC (1EK9)		−7.8482	84.1409	63.4236	39.5596	29.8075	15.9794
NorA	BCR	−4.3807	−19.3774	20.8856	14.5855	17.2122	20.5459
	CS	−9.2889	−27.7277	42.4691	14.5855	17.2122	17.3139

SBS: substrate-binding site; HT: hydrophobic trap; HH: helical hairpin; LD: lipoyl domain; BCR: binding core region; CS: cytoplasmic side.

## Data Availability

Not applicable.

## Supplementary Materials

The following supporting information can be downloaded at: https://www.mdpi.com/article/10.3390/ijms232214291/s1, Table S1. Docking results of the studied compounds in the different components of the AcrAB-TolC efflux system; Table S2. Predicted lipophilicity (expressed by Log P) of chalcones 1–10; Figure S1. Relative fluorescence curve over time of compound **3**; Figure S2. Relative fluorescence curve over time of compound **5**; Figure S3. Relative fluorescence curve over time of compound **6**; Figure S4. Relative fluorescence curve over time of compound **7**; Figure S5. Relative fluorescence curve over time of compound **8**; Figure S6. Predicted druglikeness of chalcones 1–10 for five rules (Lipinski, Ghose, Veber, Egan, and Muegge) of Medicinal Chemistry; Figure S7. Predicted value of acute toxicity in mammals (e.g., rats or mice) of chalcones 1–10 using ADMETlab 2.0.; Figure S8. ^1^H and ^13^C NMR of compound **7**; Figure S9. HRMS data of compound **7**; Figure S10. ^1^H and ^13^C NMR of compound **8**; Figure S11. HRMS data of compound **8**; Figure S12. ^1^H and ^13^C NMR of compound **9**; Figure S13. HRMS data of compound **9**; Figure S14. ^1^H and ^13^C NMR of compound **10**; Figure S15. HRMS data of compound **10**.
