# Supplementary material for: New Chalcone–Triazole Hybrids with Promising Antimicrobial Activity in Multidrug Resistance Strains
Source: Int J Mol Sci. 2022 Nov 18;23(22):14291. doi: 10.3390/ijms232214291 (PMC9697807; doi:10.3390/ijms232214291)
Supplement: Supplementary file 1 [file ijms-23-14291-s001.zip › ijms-2005197-supplementary.pdf]

Supplementary Material

New Chalcone–Triazole Hybrids with Promising Antimicrobial Activity in Multidrug Resistance Strains

Daniela Pereira [1,2,†], Fernando Durães [1,2,†], Nikoletta Szemerédi [3], Joana Freitas-da-Silva [2,4], Eugénia Pinto [2,5], Paulo Martins-da-Costa [2,4], Madalena Pinto [1,2], Marta Correia-da-Silva [1,2], Gabriella Spengler [3], Emília Sousa [1,2,*] and Honorina Cidade [1,2,*]

[1] Laboratory of Organic and Pharmaceutical Chemistry, Department of Chemical Sciences, FFUP—Faculty of Pharmacy, University of Porto, 4050-313 Porto, Portugal
[2] CIIMAR—Interdisciplinary Centre of Marine and Environmental Research, University of Porto, 4450-208 Matosinhos, Portugal
[3] Department of Medical Microbiology, Albert Szent-Györgyi Health Center and Albert Szent-Györgyi Medical School, University of Szeged, 6725 Szeged, Hungary
[4] ICBAS—Institute of Biomedical Sciences Abel Salazar, University of Porto, 4050-313 Porto, Portugal
[5] Laboratory of Microbiology, Department of Biological Sciences, Faculty of Pharmacy, University of Porto, 4050-313 Porto, Portugal
* Correspondence: esousa@ff.up.pt (E.S.); hcidade@ff.up.pt (H.C.)
† Authors contributed equally to this work and share first authorship.

Docking studies

Table S1. Docking results of the studied compounds in the different components of the AcrAB-TolC efflux system.

Compound	Docking Scores				
	AcrB		AcrA		TolC
	SBS	HT	HH	LD	
**Minocycline**	-8.7	26.7	-6.2	-5.4	-7.7
**PAβN**	-7.1	-4.7	-5.8	-4.9	-7.1
**1**	-6.8	-4.7	-6.0	-5.7	-6.6
**2**	-7.1	-3.1	-6.0	-6.2	-6.5
**3**	-8.2	1.4	-5.5	-4.8	-6.7
**4**	-9.2	1.2	-5.7	-5.1	-6.6
**5**	-8.6	4.7	-5.7	-5.0	-6.0
**6**	-7.5	-6.1	-6.6	-6.4	-6.9
**7**	-8.6	-3.9	-6.9	-6.5	-7.7
**8**	-8.4	-6.9	-7.0	-6.1	-7.4
**9**	-7.5	-6.7	-6.5	-6.3	-6.9
**10**	-7.9	-4.5	-6.1	-6.0	-7.0

Fluorescence curves

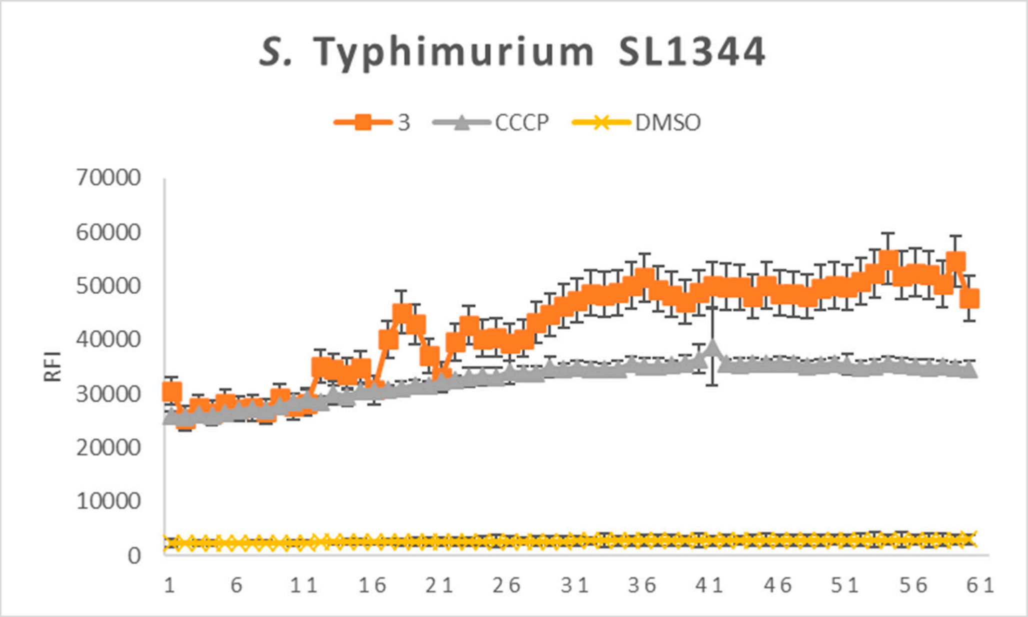

Figure S1. Relative fluorescence curve over time of compound **3**.

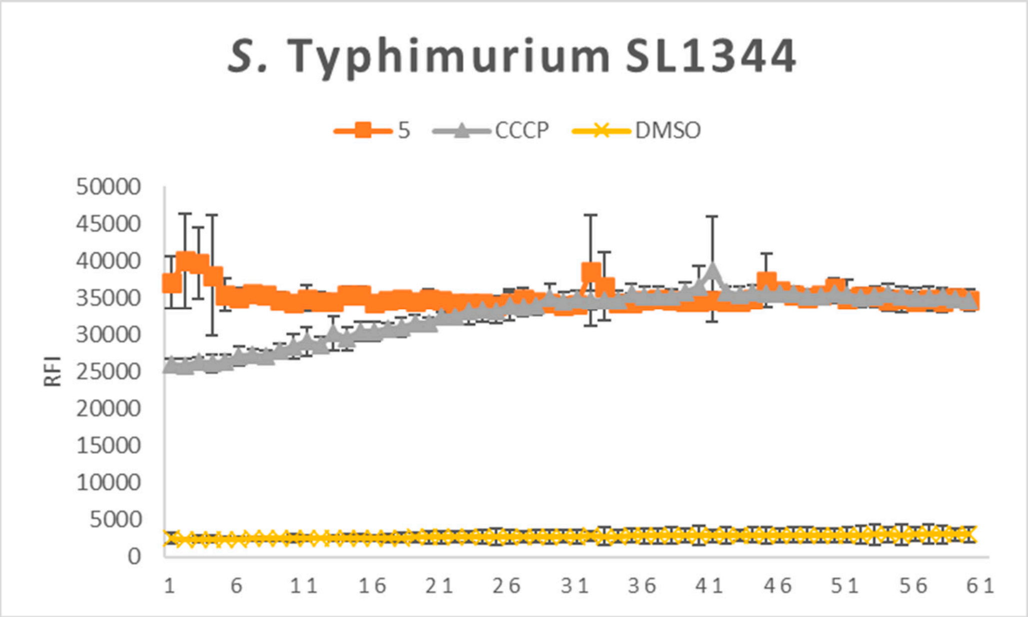

Figure S2. Relative fluorescence curve over time of compound **5**.

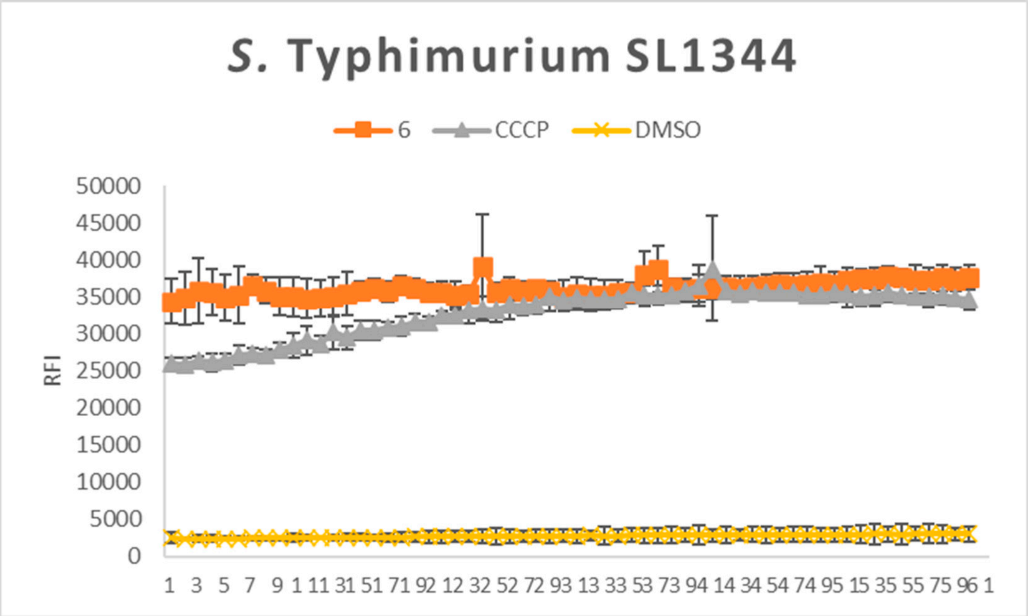

Figure S3. Relative fluorescence curve over time of compound **6**.

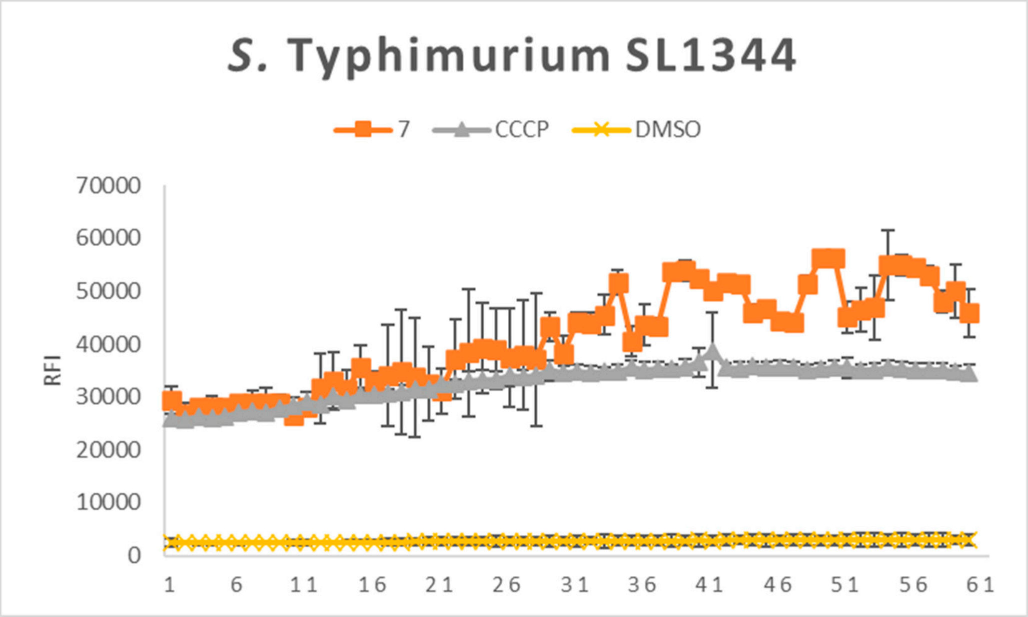

Figure S4. Relative fluorescence curve over time of compound **7**.

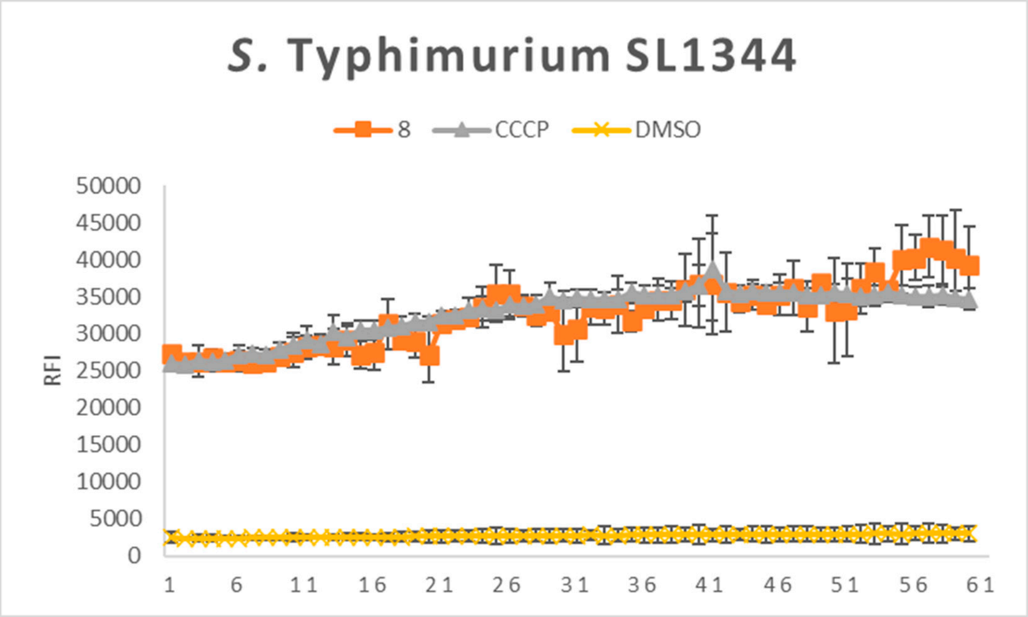

Figure S5. Relative fluorescence curve over time of compound **8**.

Log P prediction

Table S2. Predicted lipophilicity (expressed by Log P) of chalcones **1-10**.

Molecule	Log P (iLOGP)	Log P (XLOGP3)	Log P (WLOGP)	Log P (MLOGP)	Log P (SILICOS-IT)	Mean of Log P values
**1**	3.33	3.91	3.29	2.14	4.23	3.38
**2**	3.95	3.89	3.30	1.82	4.32	3.45
**3**	4.94	2.61	2.15	0.29	2.52	2.50
**4**	5.48	2.58	2.15	0.02	2.63	2.57
**5**	4.44	2.50	2.31	-0.07	2.92	2.42
**6**	5.08	2.47	2.32	-0.35	3.03	2.51
**7**	3.94	4.58	4.11	1.17	4.50	3.66
**8**	3.95	4.55	4.12	0.87	4.59	3.62
**9**	3.27	2.70	2.60	0.74	3.19	2.50
**10**	3.89	2.67	2.61	0.45	3.28	2.58
**Mean**	4.23	3.25	2.90	0.71	3.52	-

Druglikeness prediction

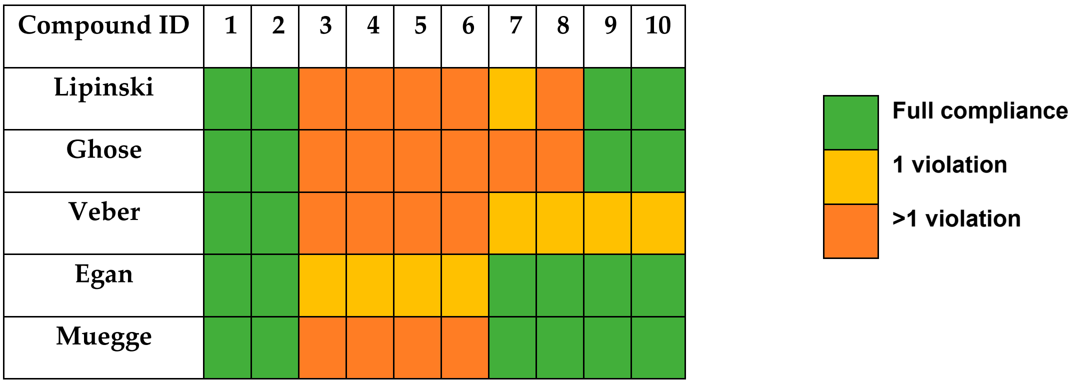

Figure S6. Predicted druglikeness of chalcones 1-10 for five rules (Lipinski, Ghose, Veber, Egan and Muegge) of Medicinal Chemistry.

Rat Oral Acute Toxicity prediction

Molecule	Rat Oral Acute Toxicity
**1**	0.3-0.7
**2**	0.3-0.7
**3**	0-0.3
**4**	0-0.3
**5**	0-0.3
**6**	0-0.3
**7**	0-0.3
**8**	0-0.3
**9**	0-0.3
**10**	0-0.3

Figure S7. Predicted value of acute toxicity in mammals (e.g. rats or mice) of chalcones **1-10** using ADMETlab 2.0. The output value range of 0 to 1 (Category 0: low-toxicity, > 500 mg/kg; Category 1: high-toxicity; < 500 mg/kg). (Green: 0-0.3; yellow: 0.3-0.7; red: 0.7-1.0)

NMR spectra of compound 7

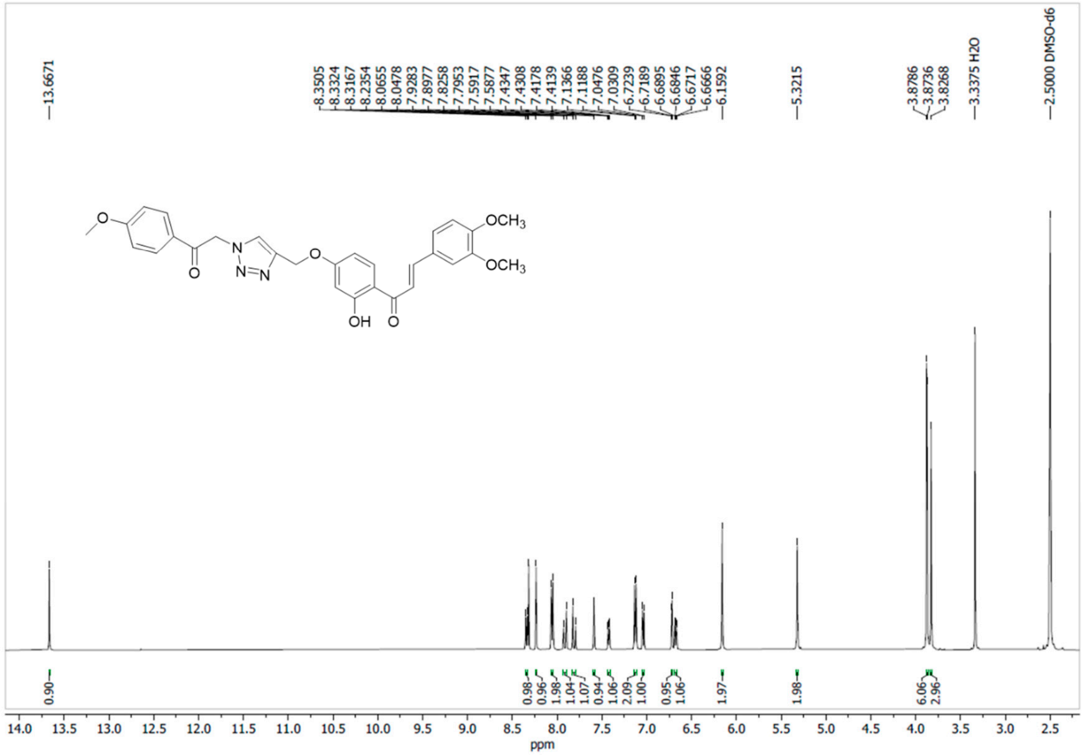

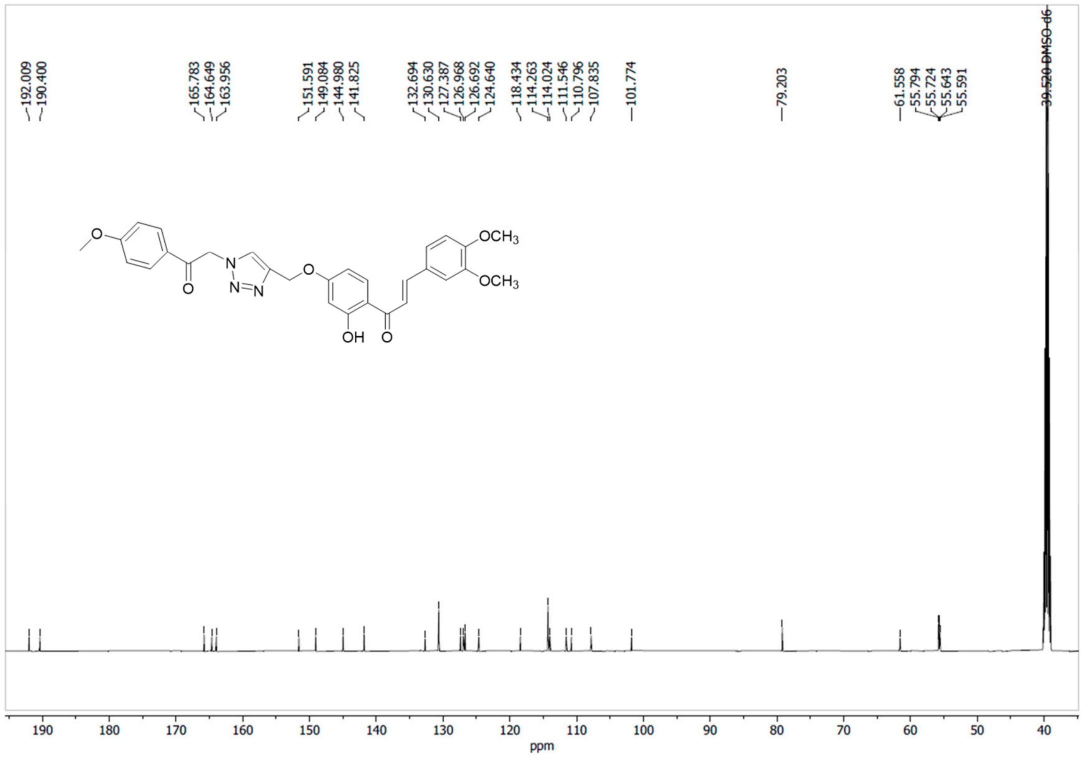

Figure S8. ^{1}H and ^{13}C NMR of compound **7**.

HRMS data of compound 7

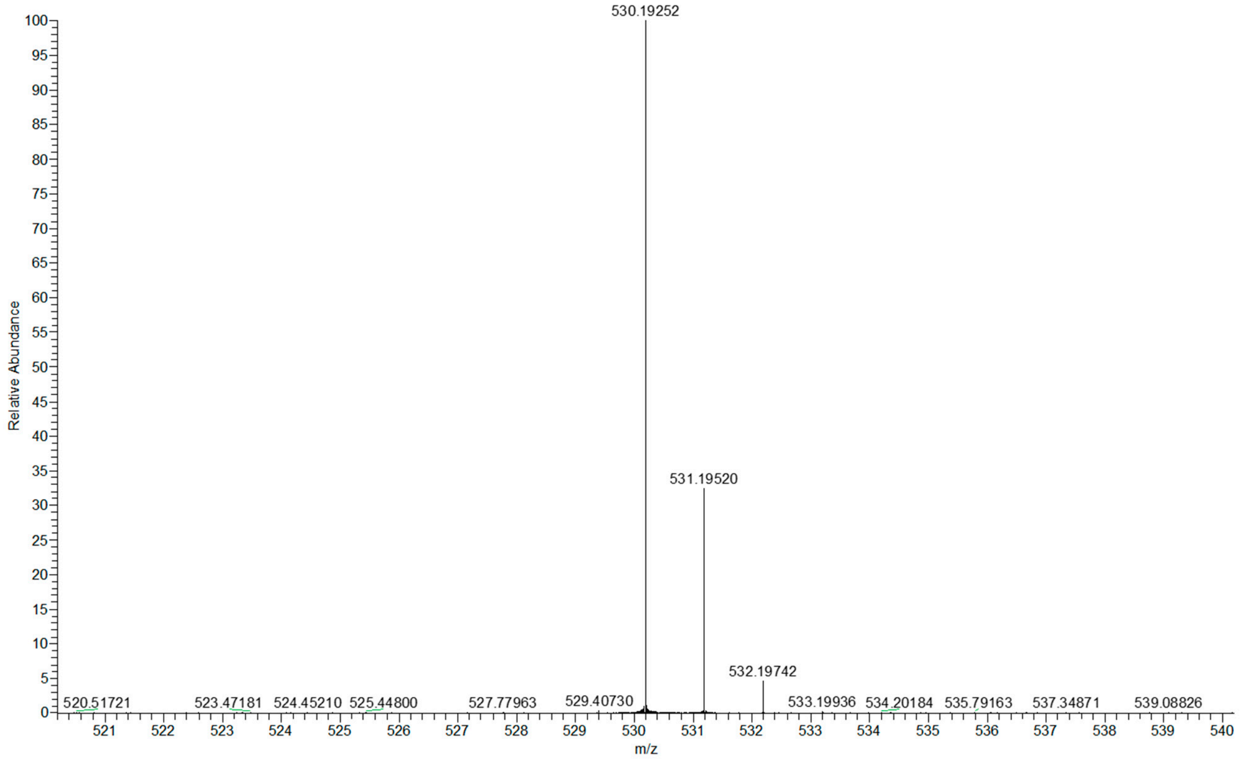

Meas. m/z	#	Ion Formula	m/z	err [ppm]	Err [mDa]
530,19252	$M+H^+$	$C_{29}H_{28}N_3O_7^+$	530,19218	0,64	0,34

Figure S9. HRMS data of compound **7**.

NMR spectra of compound 8

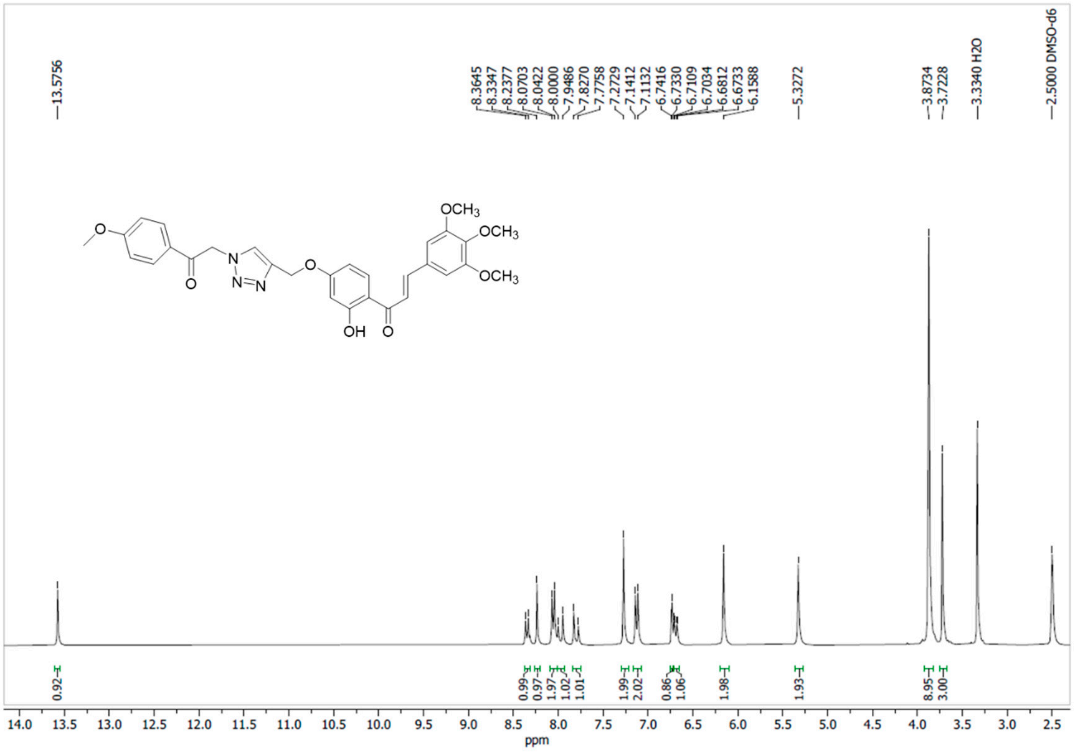

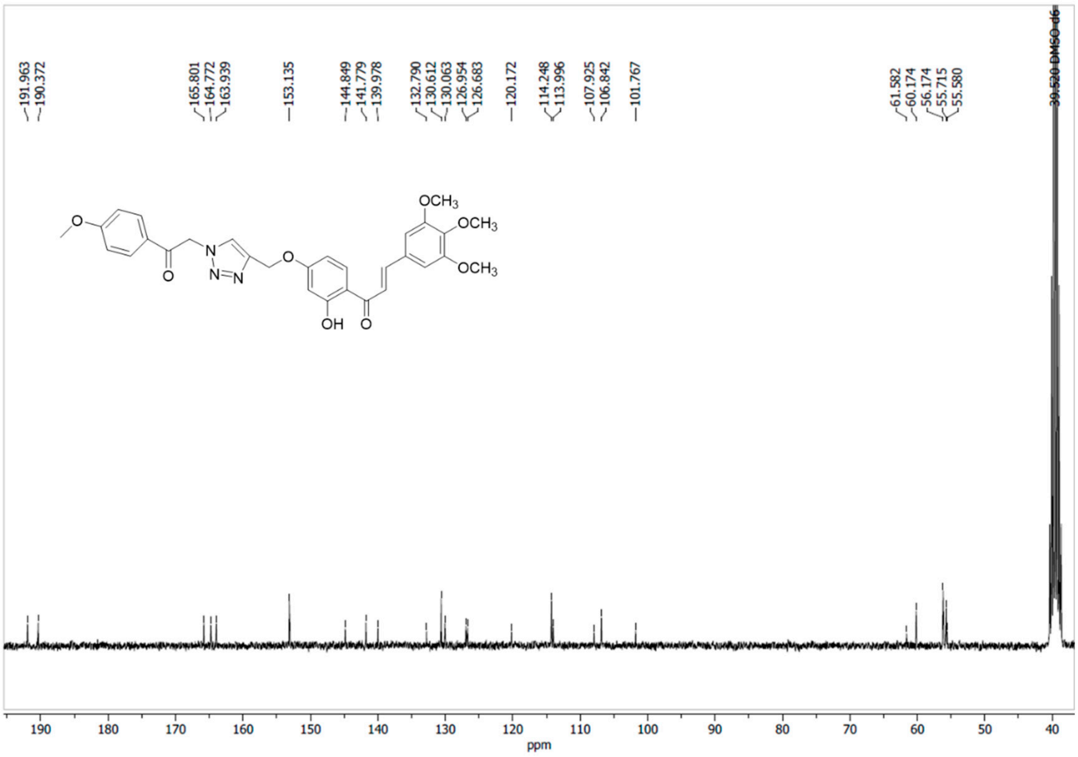

Figure S10. ^{1}H and ^{13}C NMR of compound **8**.

HRMS data of compound 8

Acquisition Parameter
Capillary Exit 300.0 V		Skimmer 1 15.0 V

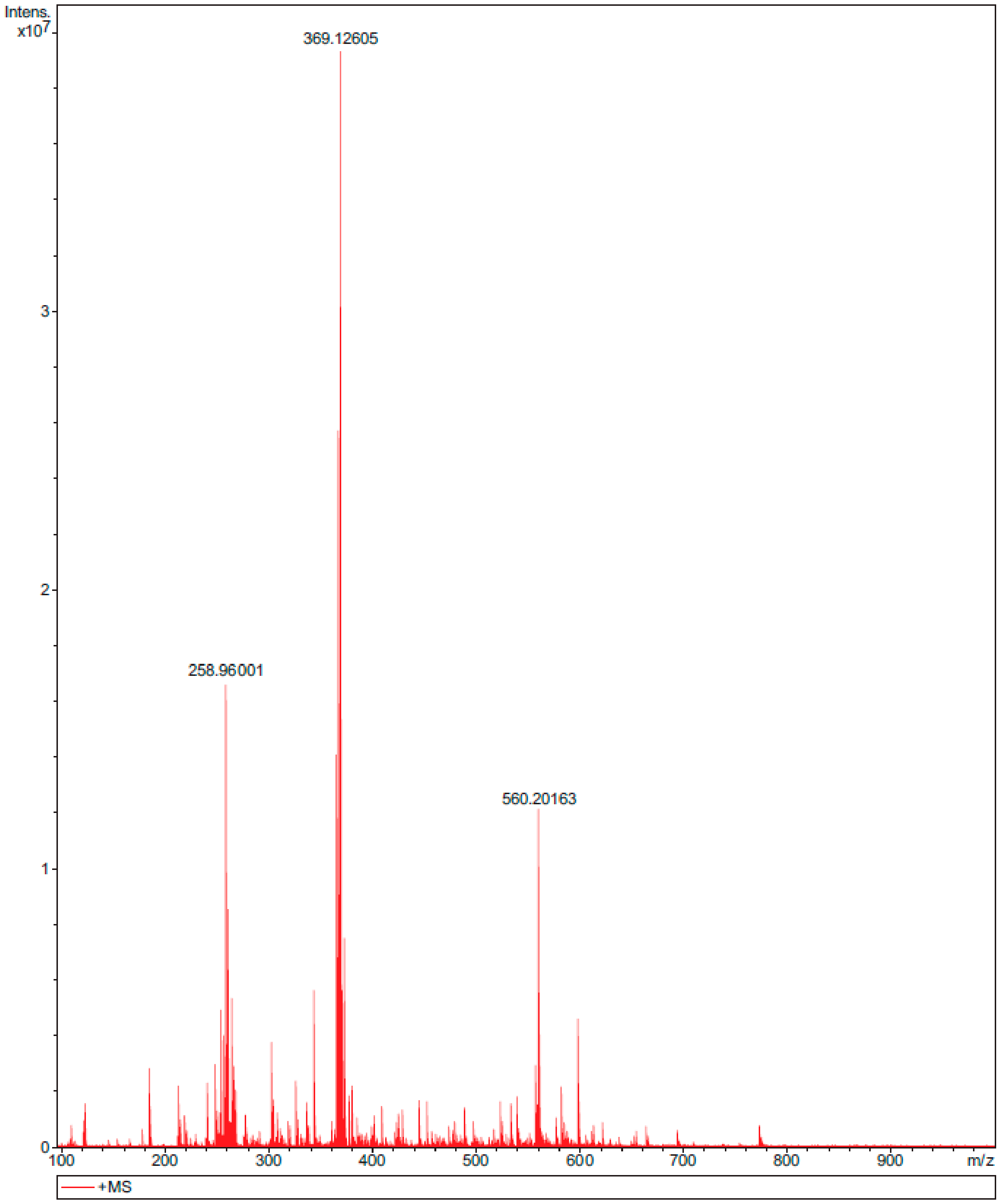

Mass Spectrum Molecular Formula Report

Meas. m/z	#	Formula	Score	m/z	err [mDa]	err [ppm]	mSigma	rdb	e⁻ Conf	N-Rule
560.20163	1	$C_{30}H_{30}N_3O_8$	100.00	560.20274	1.11	1.98	35.9	17.5	even	ok

Figure S11. HRMS data of compound **8**.

NMR spectra of compound 9

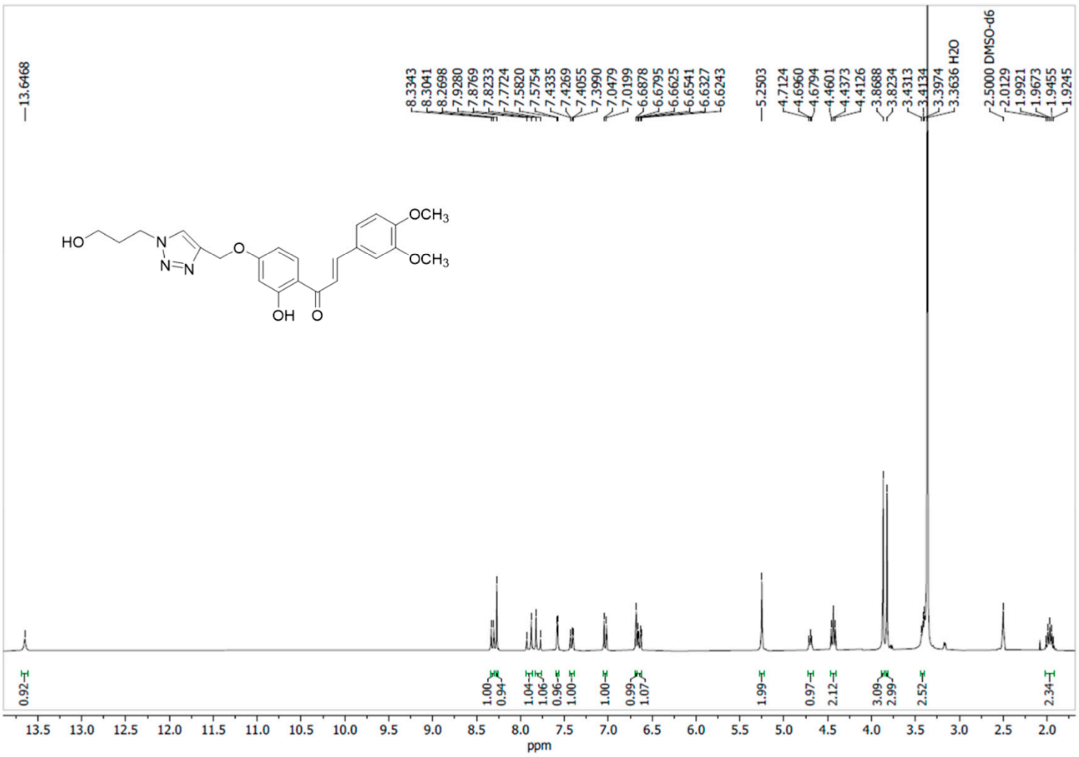

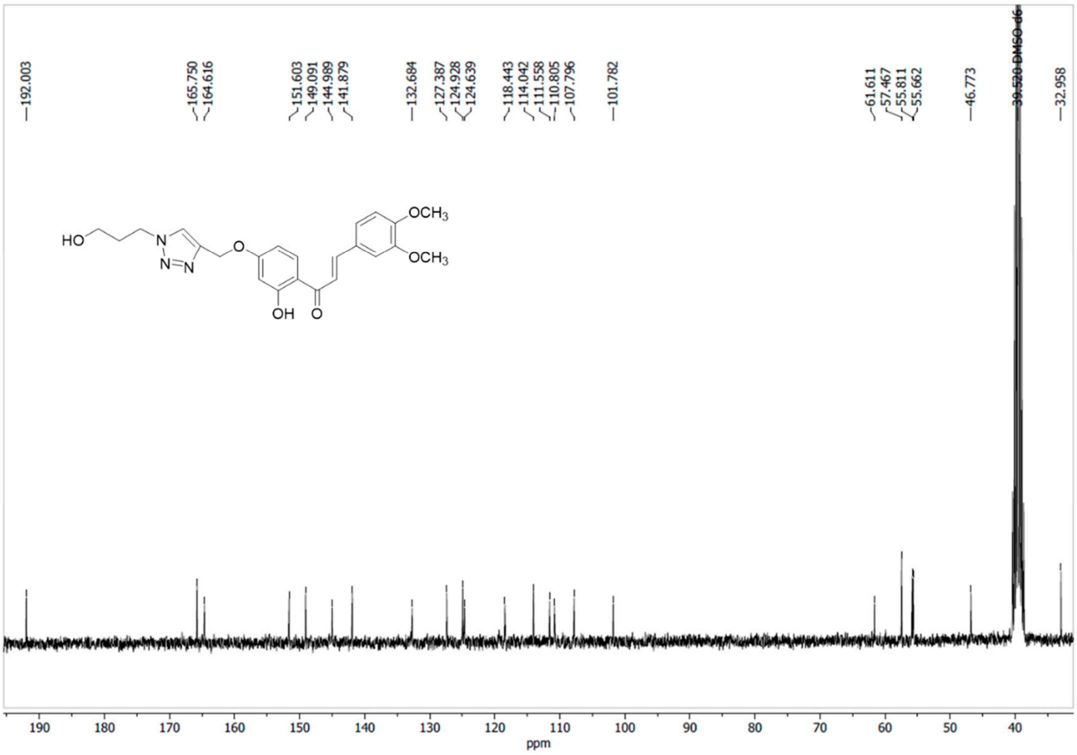

Figure S12. ^{1}H and ^{13}C NMR of compound **9**.

HRMS data of compound 9

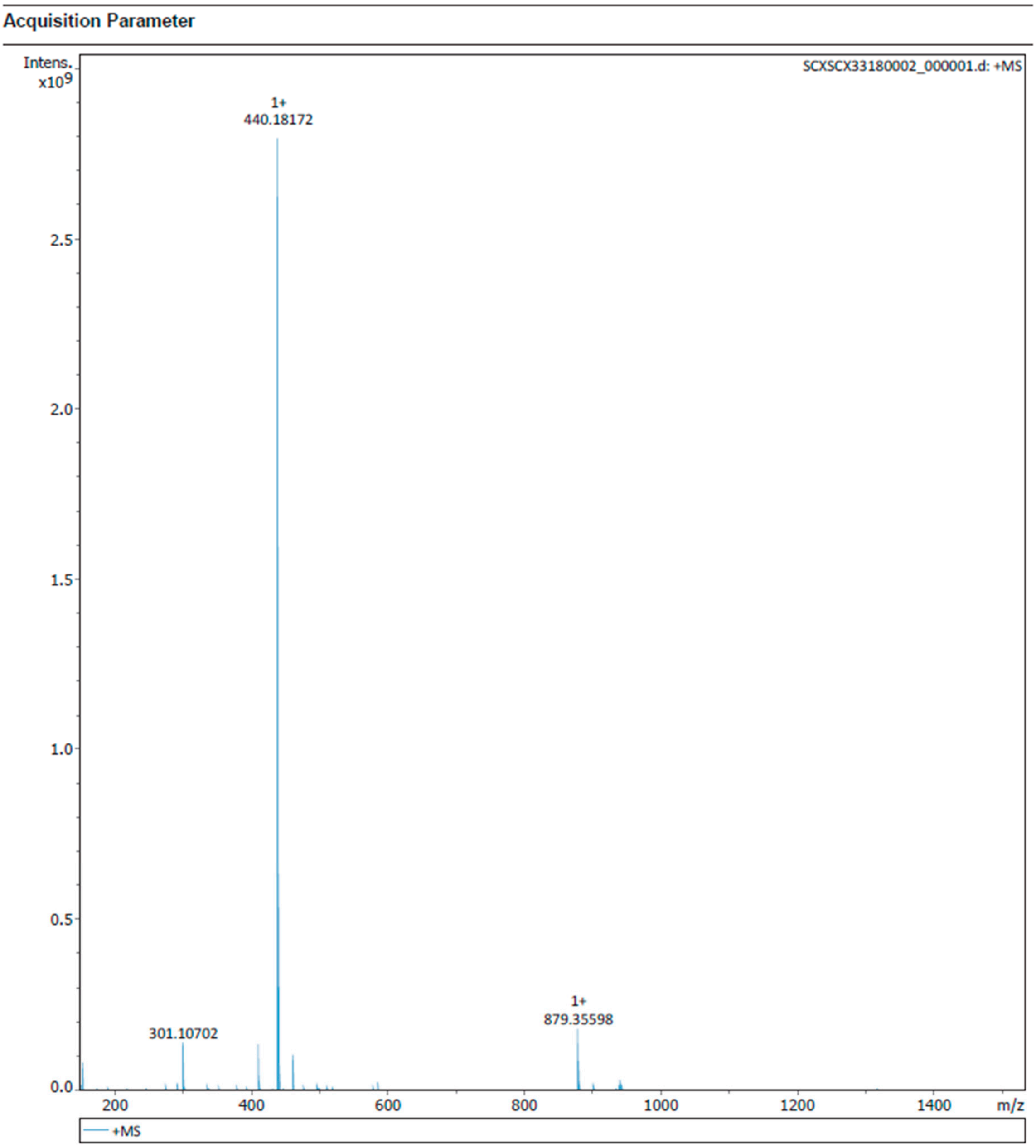

Mass Spectrum Molecular Formula Report

Meas. m/z	#	Ion Formula	Score	m/z	err [ppm]	Mean err [ppm]	mSigma	rdb	e⁻ Conf	N-Rule
440.181722	1	C23H26N3O6	100.00	440.181612	-0.3	-0.1	19.7	13.0	even	ok

Figure S13. HRMS data of compound **9**.

NMR spectra of compound 10

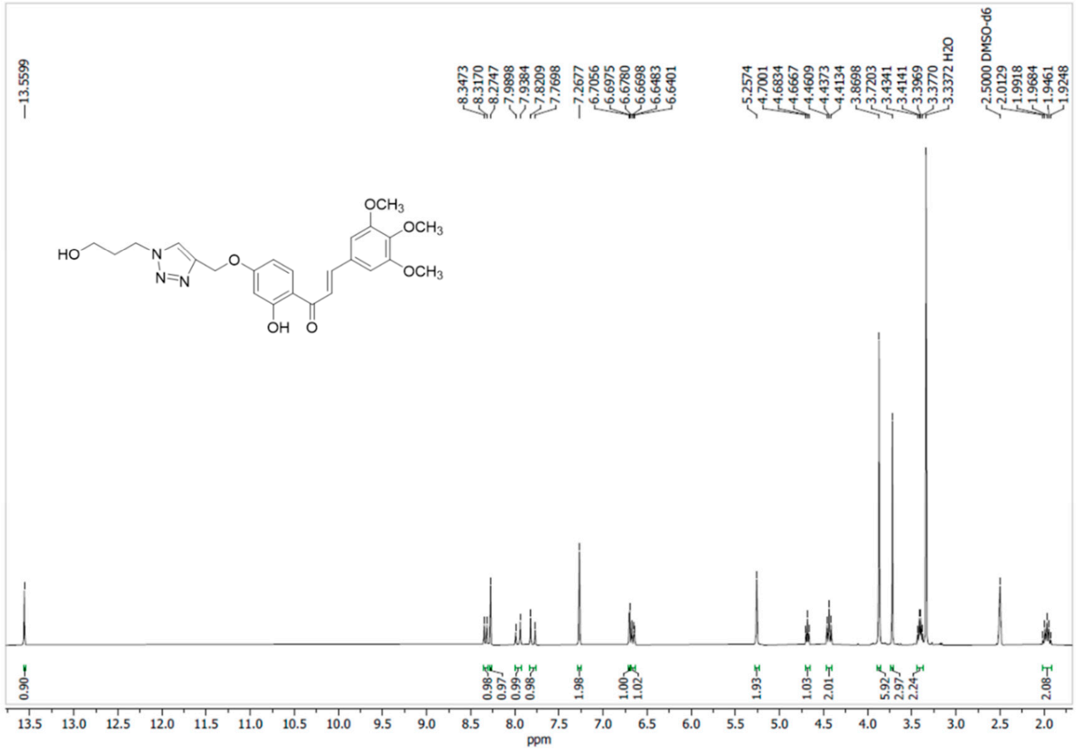

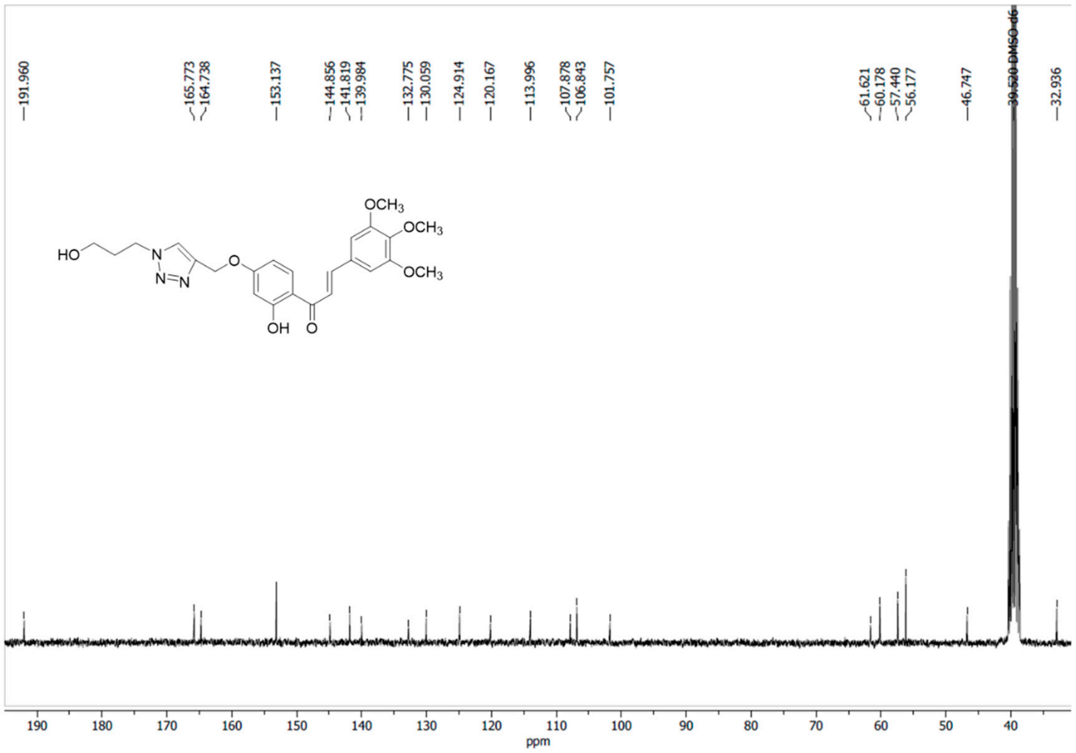

Figure S14. ^{1}H and ^{13}C NMR of compound **10**.

HRMS data of compound 10

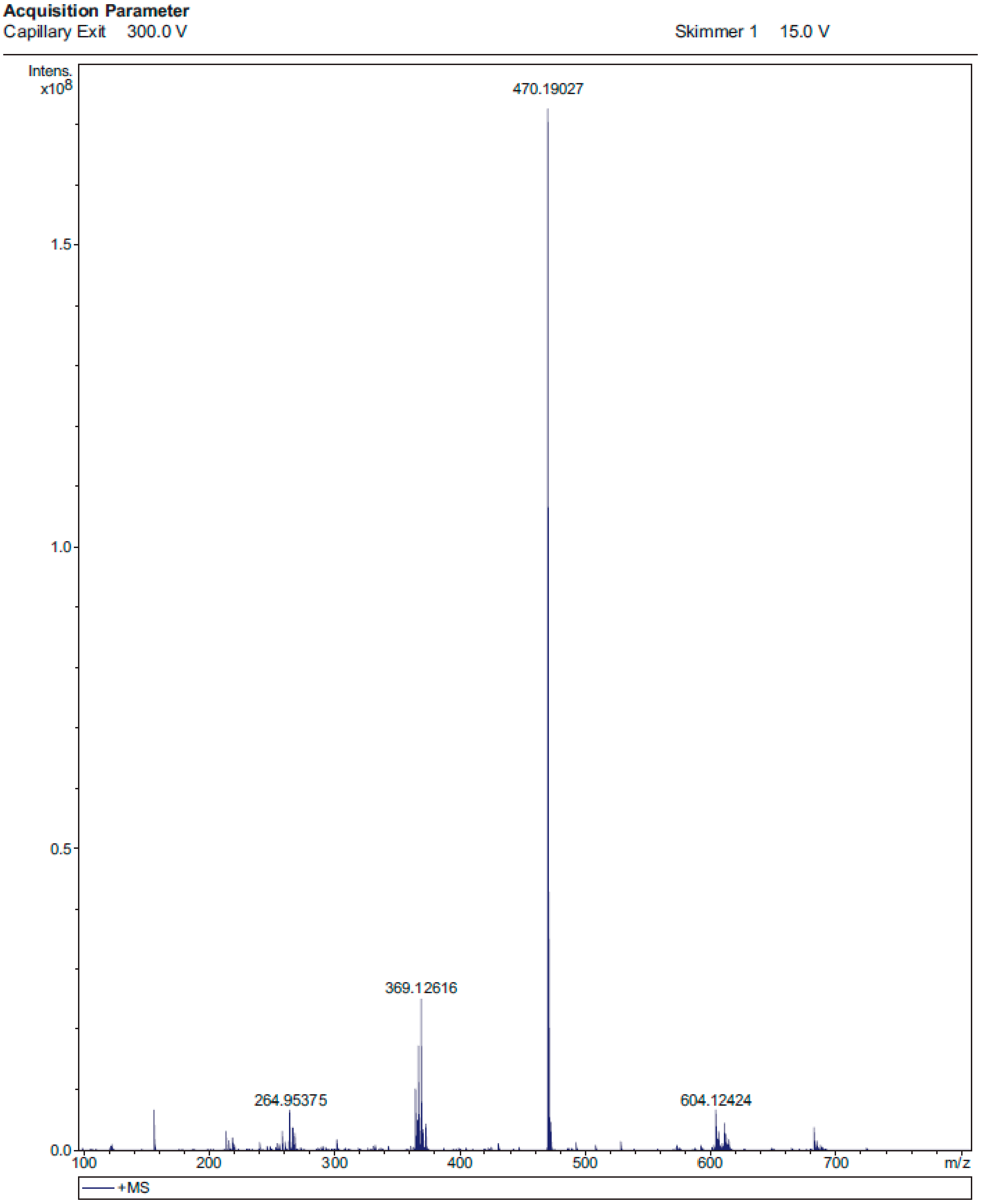

Mass Spectrum Molecular Formula Report

Meas. m/z	#	Formula	Score	m/z	err [mDa]	err [ppm]	mSigma	rdb	e⁻ Conf	N-Rule
470.19027	1	C 24 H 28 N 3 O 7	100.00	470.19218	1.90	4.05	32.4	12.5	even	ok

Figure S15. HRMS data of compound **10**.
